# Development of a warning model for drug-induced liver injury in the older patients

**DOI:** 10.3389/fphar.2025.1603089

**Published:** 2025-05-20

**Authors:** Qiaozhi Hu, Xiaoqi Li, Dan Zou, Zhiyao He, Ting Xu

**Affiliations:** ^1^ Department of Pharmacy, West China Hospital, Sichuan University, Chengdu, Sichuan, China; ^2^ West China School of Medicine, Sichuan University, Chengdu, Sichuan, China; ^3^ Key Laboratory of Drug-Targeting and Drug Delivery System of the Education Ministry, Sichuan Engineering Laboratory for Plant-Sourced Drug and Sichuan Research Center for Drug Precision Industrial Technology, West China School of Pharmacy, Sichuan University, Chengdu, Sichuan, China

**Keywords:** drug-induced liver injury, older patients, warning model, machine learning, drug safety

## Abstract

**Introduction:**

Drug-induced liver injury (DILI) is a significant adverse drug reaction, ranging from mild liver enzyme elevations to severe outcomes such as liver failure, transplantation, or death. This condition is especially concerning in older adults, who may exhibit increased susceptibility to adverse medication effects. This study aimed to develop and compare eight machine learning (ML) models using routine clinical, pharmacological, and laboratory data to predict DILI in older hospitalized patients.

**Methods:**

We conducted a retrospective analysis of older patients hospitalized in 2022 who exhibited abnormal liver function tests. A total of 421 clinical, pharmacological, and laboratory variables were utilized for model development, with missing data addressed through multiple imputation techniques. The performance of 8 ML algorithms—XGBoost, LightGBM, Random Forest, AdaBoost, CatBoost, Gradient Boosting Decision Trees, Artificial Neural Network, and TabNet—was assessed. The dataset was randomly partitioned into a training set (80%, n = 2,880) and an independent testing set (20%, n = 720). Model performance was evaluated using the area under the receiver operating characteristic curve (AUC).

**Results:**

Out of the 3,600 older patients with abnormal liver function, 654 patients experienced DILI. The best-performing model, LightGBM combined with Random Forest imputation, achieved an AUC of 0.9829. SHapley Additive exPlanations (SHAP) analysis identified critical predictors for DILI, including the timing of DILI relative to surgery, undergoing surgery, and maximum rate of change (slope) in liver enzymes, albumin, lipoprotein cholesterol, total bilirubin, proBNP, and total bile acids. Additional significant factors included administration of liver-protective medications upon admission; use of diuretics, antibiotics, and narcotic analgesics; and pre-existing liver or gallbladder diseases or malignancies.

**Discussion:**

The predictive model developed demonstrated excellent performance in identifying DILI in older adults. Leveraging machine learning techniques, this model holds significant potential for clinical implementation to effectively warn clinicians of DILI risk among older hospitalized patients.

## Introduction

Drug-induced liver injury (DILI) is a significant adverse event that can range from mild liver enzyme elevations to severe outcomes such as liver failure, the need for transplantation, or even death (Björnsson and Björnsson, 2022). DILI encompasses damage to the liver or biliary system resulting from exposure to hepatotoxic drugs (Björnsson and Björnsson, 2022). Most patients with DILI are asymptomatic, with jaundice being the most common clinical sign (Katarey and Verma, 2016). In cases of hepatocellular injury, laboratory tests reveal elevated levels of aminotransferases, such as alanine aminotransferase (ALT) and/or aspartate aminotransferase (AST), while cholestatic injury is characterized by elevations in gamma-glutamyl transferase (GGT), alkaline phosphatase (ALP), and/or bilirubin (Katarey and Verma, 2016; Kuna et al., 2018). The mechanisms of hepatotoxicity can be classified as either dose-dependent or idiosyncratic (Katarey and Verma, 2016). A substantial portion of the disease burden arises from dose-dependent toxicity, which correlates with the amount of drug exposure and is consistently reproducible, rendering liver injury in such instances predictable (Björnsson and Björnsson, 2022). Conversely, idiosyncratic liver injury is unpredictable, not directly dose-dependent, and not easily reproducible in animal models. DILI is a prevalent adverse event in clinical practice and has emerged as the leading cause of acute liver failure in the Western world (Reuben et al., 2010). Additionally, it is a primary reason for the withdrawal of medications from the market and for the issuance of safety warnings and modifications regarding drug usage (Katarey and Verma, 2016).

The aging population is characterized by the coexistence of multiple comorbid conditions, frequently leading to polypharmacy. Combined with age-related declines in physiological functions that influence drug pharmacokinetics—such as receptor sensitivity, cardiac reserve, renal function, immunological response, and homeostatic mechanisms—this significantly increases the risk of adverse drug reactions (ADRs) (Lucena et al., 2020). Consequently, older adults are considered a highly susceptible group in terms of medication safety. Prior research has identified older age as a notable risk factor for DILI (Aithal and Day, 1999; Andrade et al., 2006). For example, a Spanish study involving 882 DILI patients reported that 33% were aged 65 years or older (Weersink et al., 2021). Other contributing predictors included female sex, dyslipidemia, and diabetes (Aithal and Day, 1999; Andrade et al., 2006; Weersink et al., 2021).

As a significant ADR, DILI is actively monitored in clinical settings. Traditionally, voluntary reporting systems have been the primary means of tracking ADRs; however, they capture only 10%–20% of actual incidents, leaving the true incidence largely unknown (Griffin and Resar, 2009). Furthermore, the probability of adverse effects associated with specific drugs remains elusive, posing a significant challenge to patient safety. While active surveillance could address these limitations, it demands substantial manpower and resources, making broad implementation challenging (Hu et al., 2020). Therefore, there is an urgent need to develop innovative methods for the early detection and warning of ADRs to enhance patient safety and improve drug monitoring effectiveness.

Machine learning (ML) broadly refers to fitting models to data or identifying informative patterns within datasets (Greener et al., 2022). Essentially, ML aims to approximate human pattern recognition capabilities through objective computational methods. ML is particularly valuable when dealing with datasets that are too large or complex for human analysis, containing numerous data points or features. Furthermore, it is indispensable in automating data analysis workflows, enabling reproducible and time-efficient processes (Deo, 2015; Greener et al., 2022). Medical data often exhibit these characteristics, making ML a potent tool for disease diagnosis, detection, and prediction. Numerous studies have explored the application of ML to predict ADRs, yielding promising outcomes (Hu et al., 2024). Detecting ADRs early is equally crucial, as timely identification enables interventions that bolster patient safety and mitigate potential harm. By combining effective detection methods with predictive modeling, healthcare providers can better manage the risks associated with pharmacotherapy.

However, current DILI surveillance systems primarily rely on crude categorization of suspected cases based on abnormal liver function tests, lacking the granularity needed for definitive DILI identification. To address this limitation, we propose enhancing current systems by integrating ML algorithms trained on a cohort of patients who developed in-hospital liver dysfunction. By leveraging multimodal clinical data, our model aims to achieve precise DILI differentiation, thereby improving diagnostic accuracy and clinical decision-making. Thus, this study aimed to develop an ML algorithm for the detection and early warning of DILI, providing technical support to reduce DILI incidence. The algorithm was designed to promptly identify DILI cases, facilitating swift clinical interventions to mitigate the impact on patient health.

## Materials and methods

### Research participants

This retrospective study analyzed demographic, pharmacological, and clinical laboratory data from older patients with liver function abnormalities who were admitted to West China Hospital of Sichuan University between January 1 and December 31, 2022. Ethics approval was obtained from the Ethics Committee of West China Hospital, Sichuan University, China (Approval Number: 2022-1124). Due to the retrospective nature of the study, the requirement for informed consent was waived, and all data were fully anonymized to ensure patient confidentiality. As this study did not involve a prospective clinical trial, a clinical trial registration number was not applicable.

Eligible patients were identified according to the official Chinese definition, which classifies older adults as individuals aged 60 years and above with a minimum hospital stay of 24 h (Hu et al., 2020). Considering that clinical interventions often precede the diagnosis of DILI (Chalasani et al., 2021), patients with liver function abnormalities were identified based on liver function tests showing ALT, AST, ALP, or TBil levels exceeding 1.5 times the upper limit of normal (ULN) (Alexandre et al., 2000). The hospital’s standard ULN values for these tests were: ALT, 40 IU/L for women and 50 IU/L for men; AST, 35 IU/L for women and 40 IU/L for men; ALP, 135 IU/L for women and 160 IU/L for men; and TBil, 20.5 μmol/L for both sexes.

Eligible older patients were extracted and sorted according to their admission dates. A stratified sampling method was applied, with 150 patients randomly selected from the eligible pool every 2 weeks, ultimately yielding a total of 3,600 cases for analysis.

### Data collection and definitions

Based on previous research, the risk factors for DILI include drug exposure, individual characteristics, and genetic predispositions (Li et al., 2022). Considering these findings and the data available for collection, information was categorized into six distinct groups: demographic information, surgical data, diagnostic classification, admission status, drug details, and laboratory parameters. Demographic information, drug details, and laboratory parameters upon admission were extracted directly from the electronic medical record system.

Demographic data encompassed factors such as age, sex, marital status, allergy history, surgical history, ethnic background, smoking history, and alcohol consumption history. Given the extensive range of medications, drug information was categorized based on pharmacological effects (Wen et al., 2021). For patients without DILI, all medications administered during hospitalization were recorded. For patients with DILI, only medications administered prior to the onset of DILI were considered.

Laboratory parameters were classified into two types. The first type included stable indicators, such as viral hepatitis markers, which remain relatively constant during hospitalization and assist in identifying underlying causes of abnormal liver function. The second type comprised dynamic indicators, including liver enzymes, blood lipid levels, and cardiac function markers, which were measured multiple times during hospitalization. For these dynamic indicators, the maximum slope (i.e., the greatest rate of change) during the hospital stay was calculated and used as a feature for model development.

Surgical data, admission status, and diagnostic classification were obtained through manual review of the electronic medical records. Surgical data included whether surgery was performed, the number of surgeries, the organ(s) involved, the type of surgery, and the relationship between the timing of surgery and the onset of DILI. Admission status encompassed factors such as the method of admission, the admitting department, the nursing care level upon admission, and the number of hospitalizations in the previous year.

The diagnosis of DILI required careful manual adjudication. Hepatotoxicity was defined as elevations of ALT, AST, ALP, or TBil exceeding 1.5 times ULN, in conjunction with outcomes such as liver failure, fibrosis, cirrhosis, or death (Alexandre et al., 2000). Evaluation principles for identifying ADRs included: (1) consideration of the temporal relationship, (2) assessment of the dose-response relationship, (3) emphasis on reproducibility, (4) exclusion of alternative etiologies, and (5) recognition of known ADRs (Chapal et al., 2004; National Medical Products Administration, 2011). In this study, all principles except reproducibility were considered necessary criteria for determining the occurrence of DILI. The drug(s) most strongly implicated in causing DILI were documented.

### Data preprocessing

For demographic information, surgical data, diagnostic classification, admission status, and drug details, missing values were handled using mean imputation and random forest (RF) imputation for variables with less than 15% missingness. For the first category of laboratory parameters—those typically assessed in patients suspected of specific conditions—it was assumed that patients without corresponding test results had values within the normal range.

In contrast, for the second category of laboratory parameters, involving dynamic measures such as liver enzymes and cardiac markers, any variable with more than 30% missing data was excluded from the analysis. After preprocessing, patients were randomly stratified into a training set (80%) for model development and a testing set (20%) for model evaluation.

To further optimize model performance and address class imbalance, resampling techniques such as Random Oversampling (ROS) and the Synthetic Minority Over-sampling Technique (SMOTE) were applied.

### Construction and evaluation of multiple models

Following data preprocessing and variable selection, we developed seven machine learning models and one deep learning model: XGBoost, LightGBM, Random Forest (RF), AdaBoost, CatBoost, Gradient Boosting Decision Trees (GBDT), Artificial Neural Network (ANN), and TabNet. Hyperparameter tuning was performed using grid search, and each model was trained with 5-fold cross-validation, utilizing 20% of the training set as an internal validation set. The primary metric for evaluating and comparing model performance was the area under the receiver operating characteristic curve (AUC), which served as the principal indicator of the models’ classification capabilities.

In addition to AUC, several supplementary metrics were computed to provide a comprehensive evaluation of model performance, including accuracy, precision, sensitivity, specificity, recall, Brier score, F1 score, and average precision derived from the precision-recall curve (PRC). Calibration curves and clinical decision curve analysis (DCA) were also employed to further assess the clinical utility and calibration of the models.

To interpret the outputs of the best-performing model, SHapley Additive exPlanations (SHAP) analysis was conducted, identifying the top 50 contributing variables. In the SHAP beeswarm plot, blue points represent negative impacts (lower feature values), whereas red points represent positive impacts (higher feature values), illustrating how each feature influences the model’s predictions. SHAP waterfall plots were also used to visualize the individual contributions of variables to model outputs. Additionally, feature importance scores were calculated and presented in a dedicated figure ranking the most influential risk factors.

### Statistical analysis

Categorical variables are summarized using frequency counts and percentages, while continuous variables are presented as medians with interquartile ranges (IQRs). Comparisons between the no-DILI and DILI groups, as well as between the training and testing sets, were conducted using the nonparametric Mann-Whitney U test for continuous variables and the chi-squared (χ^2^) test for categorical variables. Statistical significance was defined as a p-value of less than 0.05. All statistical analyses were performed using SPSS version 27.0 software (IBM Corporation, Armonk, NY, United States).

## Results

### Study population

**FIGURE 1:**
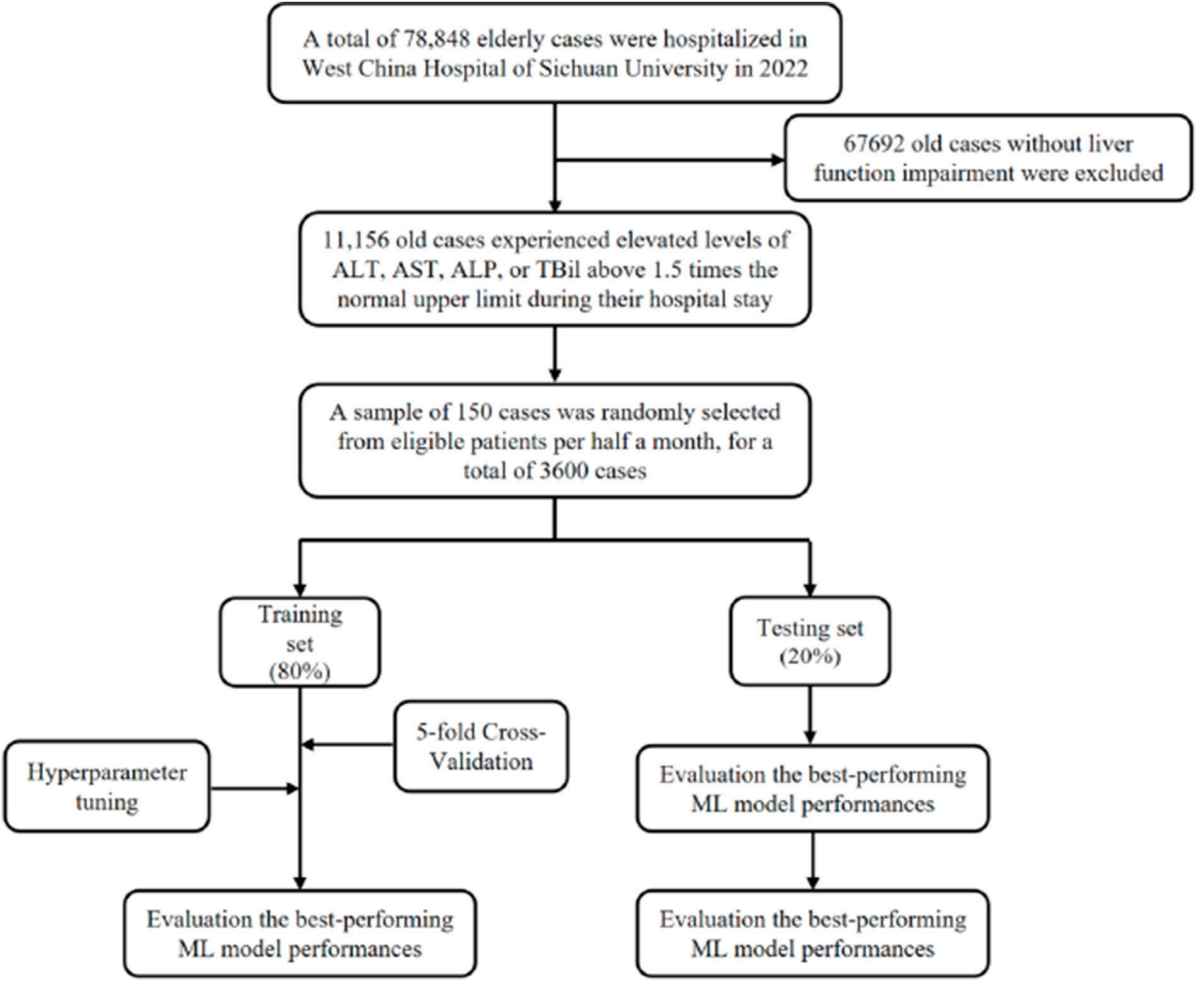
Overall flowchart of the participant selection and model development process ALT, Alanine aminotransferase; AST, Aspartate aminotransferase; ALP, Alkaline phosphatase; TBil, Total bilirubin.

A total of 11,156 older patients met the inclusion criteria, and 3,600 patients were selected for analysis following a stratified sampling method (Figure 1). Within this cohort of patients with liver function impairment, the median age was 69.00 years (IQR: 65.00–75.00), with 2,105 (58.47%) being male. Overall, 654 patients (18.17%) were diagnosed with DILI. The majority of patients were of Han nationality; 937 (26.03%) had a history of smoking, and 765 (21.25%) had a history of alcohol consumption.

Some patients were admitted primarily for liver, biliary, or pancreatic diseases, and their liver function impairment was not drug related. Consequently, there were notable differences in admission diagnoses between the DILI and non-DILI groups. Additionally, several laboratory test results were analyzed, revealing no statistically significant differences between the groups for myoglobin (Mb), creatine kinase-MB (CK-MB), albumin (ALB), high-density lipoprotein cholesterol (HDL-C), low-density lipoprotein cholesterol (LDL-C), triglycerides (TG), and cholesterol (CHO) (all P > 0.05). Detailed demographic and clinical characteristics of the two groups are presented in Table 1.

**TABLE 1:** Characteristics of included patients.

Variables (n [%] or median [IQR])	Total (n = 3,600)	Non-DILI (n = 2,946)	DILI (n = 654)	*P*
Age, years	69.00 [65.00.75.00]	69.00 [65.00.75.00]	69.00 [65.00.75.00]	0.759
Gender, n (%)				
Male	2,105 (58.47%)	1718 (58.32%)	387 (59.17%)	0.693
Female	1,495 (41.53%)	1,228 (41.68%)	267 (40.83%)	
Marital status, n (%)				
Married	3,302 (91.72%)	2,703 (91.75%)	599 (91.59%)	0.534
Single/divorced/widowed	298 (8.28%)	243 (8.25%)	55 (8.41%)	
Allergic history, n (%)				
No	3,301 (91.69%)	2,706 (91.85%)	595 (90.98%)	0.481
Yes	299 (8.31%)	240 (8.15%)	59 (9.02%)	
Ethnic groups, n (%)				
Han nationality	3,402 (94.50%)	2,784 (94.50%)	618 (95.49%)	0.668
Zang nationality	117 (3.25%)	93 (3.16%)	24 (3.67%)	
Yi nationality	26 (0.72%)	24 (0.81%)	2 (0.31%)	
Hui nationality	21 (0.58%)	17 (0.58%)	4 (0.61%)	
other nationality	34 (0.95%)	28 (0.95%)	6 (0.92%)	
Smoking history, n (%)				
No	2,663 (73.97%)	2,178 (73.93%)	485 (74.16%)	0.922
Yes	937 (26.03%)	768 (26.07%)	169 (25.84%)	
Number of cigarettes among smoking patients, n	20.00 [10.00.20.00]	20.00 [10.00.20.00]	20.00 [10.00.20.00]	0.442
Drinking history, n (%)				
No	2,835 (78.75%)	2,297 (77.97%)	538 (82.26%)	0.015
Yes	765 (21.25%)	649 (22.03%)	116 (17.74%)	
Average alcohol consumption among patients with a history of alcohol use, g	100 [100,250]	125 [95,250]	100 [100,212.5]	0.884
Surgery, n (%)				
No	1,679 (46.64%)	1,352 (45.89%)	327 (50.00%)	0.031
Yes	1921 (53.36%)	1,594 (54.11%)	327 (50.00%)	
Number of surgeries, n (%)				
0	1,679 (46.64%)	1,352 (45.89%)	327 (50.00%)	0.119
1	1859 (51.64%)	1,547 (52.51%)	312 (47.71%)	
2	58 (1.61%)	44 (1.50%)	14 (2.14%)	
3	4 (0.11%)	3 (0.10%)	1 (0.15%)	
Surgical organ, n (%)				
Total	2,247 (100%)	1908 (100%)	339 (100%)	<0.001
Heart/Aorta	422 (18.78%)	359 (18.81%)	63 (18.58%)	
Brain	127 (5.65%)	58 (1.78%)	69 (10.36%)	
Peripheral Vessels	15 (0.67%)	13 (0.40%)	2 (0.30%)	
Liver	568 (25.28%)	567 (17.39%)	1 (0.15%)	
Biliary, Pancreas or Spleen	431 (19.18%)	427 (13.98%)	4 (0.60%)	
Chest (Lungs or Mediastinum)	118 (5.25%)	66 (2.02%)	52 (7.81%)	
Digestive System, Abdominal Cavity (Other than Liver, Biliary, Pancreas, Spleen)	271 (12.06%)	211 (6.47%)	60 (9.01%)	
Skeleton (Limbs or Spine)	151 (6.72%)	105 (3.22%)	46 (6.91%)	
Kidneys and Urinary System	72 (3.20%)	46 (1.41%)	26 (3.90%)	
Others Organ	72 (3.20%)	56 (1.72%)	16 (2.40%)	
Disease, n				
Total	5,363 (100.00%)	4,849 (100.00%)	512 (100.00%)	<0.001
Malignant tumors (excluding liver, pancreas, gallbladder)	790 (14.73%)	618 (12.74%)	172 (33.59%)	
Liver cancer or liver metastatic cancer	676 (12.60%)	673 (13.88%)	3 (0.59%)	
Pancreatic cancer or pancreatic metastatic cancer	141 (2.63%)	141 (2.91%)	0 (0.00%)	
Gallbladder cancer or gallbladder metastatic cancer	118 (2.20%)	116 (2.39%)	2 (0.39%)	
Bone tumors or metastatic tumors	75 (1.40%)	70 (1.44%)	5 (0.98%)	
Viral/Alcoholic liver disease/Liver cirrhosis/Other liver diseases	1,164 (21.70%)	1,128 (23.26%)	34 (6.64%)	
Acute cerebral infarction or intracranial hemorrhage (excluding malignant tumors)	189 (3.52%)	116 (2.92%)	73 (14.26%)	
Gallbladder diseases (excluding malignant tumors)	535 (9.98%)	525 (10.83%)	10 (1.95%)	
Pancreatic diseases (excluding malignant tumors)	143 (2.67%)	140 (2.89%)	3 (0.59%)	
AECOPD/respiratory failure	281 (5.24%)	228 (4.70%)	53 (10.35%)	
Chronic renal insufficiency or renal failure	148 (2.76%)	124 (2.56%)	24 (4.69%)	
Bone diseases (excluding malignant tumors)	159 (2.96%)	136 (2.80%)	23 (4.49%)	
Acute coronary syndrome or heart disease or heart failure or cardiac arrest	266 (4.96%)	240 (4.95%)	26 (5.08%)	
Heart valve disease (moderate/severe)	349 (6.51%)	310 (6.39%)	39 (7.62%)	
Venous embolism	55 (1.02%)	41 (0.84%)	14 (2.73%)	
Shock	94 (1.75%)	82 (1.69%)	12 (2.34%)	
Aortic dissection or aneurysm	75 (1.40%)	64 (1.32%)	11 (2.15%)	
Peritonitis/abdominal cavity infection	105 (1.96%)	97 (2.00%)	8 (1.56%)	
Medical history in the 10 days prior to this hospitalization, n (%)				
Yes	1,273 (35.36%)	1,046 (35.51%)	227 (34.71%)	0.700
No	2,327 (64.64%)	1900 (64.49%)	427 (65.29%)	
Number of hospitalizations in this hospital in the past year, n (%)				
0	2,756 (76.56%)	2,230 (75.70%)	526 (80.43%)	0.059
1–5	752 (20.89%)	641 (21.76%)	111 (16.97%)	
6–10	75 (2.08%)	61 (2.07%)	14 (2.14%)	
10-	17 (0.47%)	14 (0.47%)	3 (0.46%)	
Level of nursing care upon admission, n (%)				
Specialized nursing care	183 (5.09%)	148 (5.02%)	35 (5.35%)	0.303
Primary care	1933 (53.69%)	1,566 (53.16%)	367 (56.12%)	
Secondary care	1,484 (41.22%)	1,232 (41.82%)	252 (38.53%)	
Type of admission, n (%)				
Emergent	1,573 (43.69%)	1,283 (43.55%)	290 (44.34%)	0.712
Elective	2027 (56.31%)	1,663 (56.45%)	364 (55.66%)	
Method of admission, n (%)				
On foot	2,167 (60.19%)	1783 (60.52%)	384 (58.72%)	0.660
Wheel chair	140 (3.89%)	115 (3.9%)	25 (3.82%)	
Gurney	1,293 (35.92%)	1,048 (35.57%)	245 (37.46%)	
Initial Laboratory Test Results at Admission				
Pro-BNP (ng/L)	632.5 [163.75,2345.75]	695 [163,2636.75]	474 [167.75,1456.5]	0.005
Mb (ng/mL)	48.49 [28.19,150.00]	48.01 [27.87,161.50]	49.54 [28.88,127.20]	0.640
CK-MB (ng/mL)	1.73 [0.98.3.35]	1.74 [0.99.3.46]	1.69 [0.97.3.02]	0.094
TnT (ng/L)	17.60 [10.50.46.50]	18.10 [10.60.51.60]	16.20 [10.10.30.68]	<0.001
ALT (IU/L)	37.00 [19.00.78.00]	42.00 [20.00.56.00]	24.00 [15.00.46.00]	<0.001
AST (IU/L)	43.00 [24.00.79.00]	50.00 [27.00.87.00]	19.00 [26.00.40.00]	<0.001
Alb (g/L)	37.65 [32.60.37.65]	37.60 [32.50.42.20]	37.90 [33.00.42.10]	0.519
ALP (IU/L)	107.00 [76.00,196.00]	117.00 [80.00,224.00]	81.00 [65.00,106.25]	<0.001
GGT (IU/L)	61.00 [26.00,175.00]	74.00 [30.00,204.00]	33.00 [18.00.68.00]	<0.001
HDL-C (mmol/L)	1.06 [0.78.1.36]	1.06 [0.76.1.36]	1.07 [0.82.1.37]	0.176
LDL-C (mmol/L)	2.09 [1.42.2.78]	2.08 [1.41, 2.76]	2.13 [1.48.2.83]	0.161
TG (mmol/L)	1.23 [0.91.1.73]	1.75 [1.24, 2.53]	1.21 [1.21.2.35]	0.097
CHO (mmol/L)	3.86 [3.05.4.77]	3.86 [3.05.4.78]	3.85 [3.05.4.76]	0.389
TBA (umol/L)	5.95 [2.90.14.22]	6.70 [3.30.17.00]	3.50 [2.10.6.70]	<0.001
TBil (umol/L)	13.60 [9.20.23.17]	14.40 [9.50.26.13]	11.20 [8.00.15.90]	<0.001
DBil (umol/L)	5.00 [3.20.10.00]	5.40 [3.40.11.83]	3.70 [2.60.5.60]	<0.001
IBil (umol/L)	8.10 [5.40.12.30]	8.30 [5.50.13.00]	7.15 [4.97.10.00]	<0.001
AFP (ng/mL)	2.86 [1.85.4.37]	2.91 [1.90, 4.53]	2.43 [1.67.3.75]	<0.001
Viral hepatitis laboratory test results				
HAV-IgG (S/CO)	0.02 [0.01.0.08]	0.02 [0.01.0.08]	0.04 [0.01.0.13]	0.445
HBsAb semi-quant (IU/L)	23.52 [2.00,128.75]	20.10 [2.00,120.00]	41.40 [3.77,195.00]	<0.001
HBsAg semi-quant (COI)	0.43 [0.39.0.50]	0.43 [0.39.0.51]	0.42 [0.38.0.47]	<0.001
HBeAb semi-quant (COI)	1.18 [0.68.1.45]	1.16 [0.61.1.44]	1.24 [0.97.1.48]	<0.001
HBeAg semi-quant (COI)	0.09 [0.08.0.10]	0.09 [0.08.0.10]	0.09 [0.08.0.10]	0.607
HBcAb semi-quant (COI)	0.01 [0.01.0.75]	0.01 [0.01.0.72]	0.02 [0.01.1.03]	0.032
HP-HBV VL (IU/mL)	0.00 [0.00,192.75]	0.00 [0.00,182.75]	27.35 [0.00,17,116.50]	0.293
anti-HCV (COI)	0.04 [0.04.0.04]	0.04 [0.04.0.05]	0.04 [0.04.0.04]	0.006
HP-HCV VL (IU/mL)	0.00 [0.00,940,500.00]	0.00 [0.00,968,250]	0.00 [0.00,940,500.00]	0.552
HDV-IgM (S/CO)	0.05 [0.03.0.08]	0.05 [0.03.0.07]	0.08 [0.06, 0.08]	0.200
HEV-IgG (S/CO)	0.68 [0.07.8.75]	0.51 [0.04.7.60]	3.09 [0.09, 12.77]	0.501
HEV-IgM (S/CO)	0.03 [0.02.0.13]	0.03 [0.02.0.13]	0.04 [0.02, 0.11]	0.859

Abbreviations: AECOPD, acute exacerbation of chronic obstructive pulmonary disease; Pro-BNP, Pro-Brain Natriuretic Peptide; Mb, Myoglobin; CK-MB, Creatine Kinase-MB; TnT, Troponin T; ALT, alanine aminotransferase; AST, aspartate aminotransferase; Alb, Albumin; ALP, alkaline phosphatase; GGT, Gamma-Glutamyl transferase; HDL-C, High-Density Lipoprotein Cholesterol; LDL-C, Low-Density Lipoprotein Cholesterol; TG, triglycerides; CHO, cholesterol; TBA, total bile acids; TBil, Total Bilirubin; DBil, Direct Bilirubin; IBil, Indirect Bilirubin; AFP, Alpha-Fetoprotein; HAV-IgG, Hepatitis A Virus Immunoglobulin G; HBsAb semi-quant, Hepatitis B Surface Antibody semi-quantitative; HBsAg semi-quant, Hepatitis B Surface Antigen semi-quantitative; HBeAb semi-quant, Hepatitis B e Antibody semi-quantitative; HBeAg semi-quant, Hepatitis B e Antigen semi-quantitative; HBcAb semi-quant, Hepatitis B Core Antibody semi-quantitative; HP-HBV VL, High Precision Hepatitis B Virus Viral Load; anti-HCV, Antibody to Hepatitis C Virus; HP-HCV VL, High Precision Hepatitis C Virus Viral Load; HDV-IgM, Hepatitis D Virus Immunoglobulin M; HEV-IgG, Hepatitis E Virus Immunoglobulin G; HEV-IgM, Hepatitis E Virus Immunoglobulin M. note, For categorical variables, frequencies and percentages are reported. For continuous variables, data are presented as median and interquartile range [IQR] for non-normally distributed data. P values <0.05 were considered statistically significant.

The patients were randomly stratified into a training set (80%) and a testing set (20%). A comprehensive comparison of demographic and clinical laboratory characteristics between the training and testing sets is provided in Supplementary Table S1. No statistically significant differences were observed in most demographic and clinical characteristics between the two groups (all P > 0.05), indicating that the random stratification achieved a well-balanced distribution. Minor observed differences were considered within an acceptable tolerance threshold.

### DILI-related drugs

Among the 654 patients who developed DILI, a total of 1,036 drugs from 38 different classes were identified as potential causative agents. Notably, some cases of DILI involved multiple drugs, complicating the attribution to a single causative agent. Antibiotics were the most frequently implicated class, associated with DILI in 380 patients. Following antibiotics, anticoagulants (136 patients), antineoplastic agents (39 patients), and antipyretic, analgesic, and anti-inflammatory drugs (65 patients) were also commonly involved.

Other frequently implicated drug classes included medications for peptic ulcers and gastroesophageal reflux disease, antifungal agents, lipid-regulating and anti-atherosclerotic agents, gastrointestinal motility drugs, antiemetics, and antiepileptic agents. Among hospitalized patients with liver function impairment, the highest DILI incidence rates were observed for antidepressants (15.38%), antituberculosis drugs (14.63%), antimicrobial agents (14.55%), antifungal agents (12.33%), and antiepileptic agents (10.04%). Detailed information on DILI-related drugs is presented in Supplementary Table S2.

### Construction and comparison of multiple ML models

A total of 421 variables were utilized for model development (Supplementary Table S3). Several variables required imputation for missing values, including height, weight, the number of cigarettes smoked by patients who smoke, the duration of smoking among smokers, average alcohol consumption among drinkers, the duration of drinking among drinkers, and glycated hemoglobin (HbA1c) levels. Supplementary Table S4 provides a comparison of data before and after imputation.

Seven machine learning (ML) models and one deep learning model were employed: XGBoost, LightGBM, RF, AdaBoost, CatBoost, GBDT, ANN, and TabNet. To ensure accuracy and model stability, 5-fold cross-validation (CV) and grid search were used for hyperparameter tuning. The optimal hyperparameters for each model are detailed in Supplementary Table S5.

The AUC was the primary metric used to evaluate model performance. After imputing missing values using the RF method, the LightGBM model demonstrated the best performance, achieving an AUC of 0.9829 in the testing set. Attempts to further improve model performance through resampling techniques were unsuccessful, with resampling yielding suboptimal results. Additional evaluation metrics, including accuracy, sensitivity, specificity, precision, Brier score, and F1 score for the testing set, are summarized in Table 2. The detailed performance results of the ML models are presented in Supplementary Figures S1–S6.

**TABLE 2:** Training and testing set results of the machine-learning models.

Model	Missing value imputation and resampling	Accuracy	Sensitivity	Specificity	Precision	F1 score	Brier score	AUC (test set)	AUC (train set)
AdaBoost	Mean Imputation-no resampling	0.9431	0.7091	0.9852	0.8965	0.7919	0.1829	0.9752	0.9863
CatBoost		0.9431	0.7364	0.9803	0.8710	0.7980	0.0397	0.9757	0.9994
GDBT		0.9492	0.7879	0.9754	0.83871	0.8125	0.0444	0.9706	0.9992
LightGBM		**0.9556**	**0.8000**	**0.9836**	**0.8980**	**0.8461**	**0.0363**	**0.9819**	**1.0000**
XGBoost		0.9514	0.8182	0.9754	0.8571	0.8372	0.0387	0.9788	1.0000
RF		0.9319	0.5636	0.9984	0.9841	0.7168	0.0609	0.9534	1.0000
ANN		0.8625	0.4636	0.9344	0.5604	0.5075	0.1016	0.8593	0.9898
TabNet		0.8472	0.0000	1.0000	0.0000	0.0000	0.1286	0.7578	0.7878
AdaBoost	RF Imputation-no resampling	0.9361	0.8273	0.9557	0.7712	0.7982	0.2258	0.9786	0.9998
CatBoost		0.9389	0.6818	0.9852	0.8929	0.7732	0.0413	0.9747	0.9975
GDBT		0.9417	0.7364	0.9787	0.8617	0.7941	0.0417	0.9732	1.0000
LightGBM		**0.9542**	**0.8000**	**0.9820**	**0.8889**	**0.8421**	**0.0381**	**0.9829**	**1.0000**
XGBoost		0.9458	0.7636	0.9787	0.8660	0.8116	0.0397	0.9775	1.0000
RF		0.9319	0.5636	0.9984	0.9841	0.7168	0.0630	0.9527	1.0000
ANN		0.8583	0.4727	0.9279	0.5417	0.5048	0.1077	0.8529	0.9905
TabNet		0.9167	0.4545	1.0000	1.0000	0.6250	0.0728	0.8905	0.8746
AdaBoost	Mean Imputation-ROS	0.9542	0.9847	0.4219	0.9674	0.9760	0.2358	0.8431	0.9934
CatBoost		**0.9712**	**0.9991**	**0.4844**	**0.9712**	**0.9850**	**0.0264**	**0.9030**	**0.9998**
GDBT		0.9661	0.9910	0.5312	0.9736	0.9822	0.0309	0.8712	1.0000
LightGBM		0.9720	1.0000	0.4844	0.9712	0.9854	0.0260	0.8907	1.0000
XGBoost		0.9737	1.0000	0.5156	0.9729	0.9863	0.0254	0.8752	1.0000
RF		0.9720	1.0000	0.4844	0.9712	0.9854	0.0265	0.9024	1.0000
ANN		0.9627	0.9928	0.4375	0.9685	0.9805	0.0338	0.8078	0.9763
TabNet		0.9576	0.9928	0.3437	0.9634	0.9779	0.0407	0.7712	0.8732
AdaBoost	RF Imputation-ROS	0.9567	0.9839	0.4844	0.9708	0.9773	0.2354	0.8387	0.9934
CatBoost		0.9729	1.0000	0.5000	0.9721	0.9858	0.0255	0.8905	0.9999
GDBT		0.9644	0.9901	0.5156	0.9727	0.9813	0.0320	0.8631	1.0000
LightGBM		0.9737	1.0000	0.5156	0.9729	0.9863	0.0256	0.8945	1.0000
XGBoost		0.9737	1.0000	0.5156	0.9729	0.9863	0.0257	0.8957	1.0000
RF		**0.9720**	**1.0000**	**0.4844**	**0.9712**	**0.9854**	**0.0264**	**0.9022**	**1.0000**
ANN		0.9678	0.9964	0.4687	0.9703	0.9832	0.0297	0.8004	0.9837
TabNet		0.9457	1.0000	0.0000	0.9457	0.9721	0.0511	0.5953	0.5972
AdaBoost	Mean Imputation-SMOTE	0.9347	0.9864	0.2152	0.9459	0.9657	0.2370	0.7972	0.9754
CatBoost		0.9533	1.0000	0.3038	0.9524	0.9756	0.0398	0.8362	0.9997
GDBT		0.9516	0.9964	0.3291	0.9539	0.9746	0.0419	0.8213	1.0000
LightGBM		0.9593	0.9991	0.4051	0.9590	0.9786	0.0388	0.8572	1.0000
XGBoost		**0.9559**	**0.9982**	**0.3671**	**0.9564**	**0.9769**	**0.0393**	**0.8599**	**1.0000**
RF		0.9491	0.9991	0.2532	0.9490	0.9734	0.0440	0.8296	1.0000
ANN		0.9491	0.9845	0.4557	0.9618	0.9730	0.0443	0.7717	0.9878
TabNet		0.9313	0.9982	0.0000	0.9329	0.9644	0.0636	0.6164	0.5650
AdaBoost	RF Imputation-SMOTE	0.9279	0.9782	0.2278	0.9463	0.9620	0.2313	0.7982	0.9423
CatBoost		0.9550	1.0000	0.3291	0.9540	0.9765	0.0385	0.8463	0.9995
GDBT		0.9516	0.9927	0.3797	0.9570	0.9746	0.0440	0.7955	1.0000
LightGBM		0.9584	0.9973	0.4177	0.9597	0.9781	0.0374	0.8814	1.0000
XGBoost		**0.9576**	**0.9991**	**0.3797**	**0.9573**	**0.9778**	**0.0378**	**0.8832**	**1.0000**
RF		0.9500	0.9991	0.2658	0.9499	0.9739	0.0425	0.8594	1.0000
ANN		0.9584	0.9936	0.4683	0.9630	0.9781	0.0387	0.8046	0.9876
TabNet		0.9296	0.9936	0.0380	0.9350	0.9634	0.0613	0.7542	0.7290

Abbreviations: AdaBoost, Adaptive Boosting; CatBoost, Categorical Boosting; GDBT, gradient boosting decision tree; LightGBM, light gradient boosting machine; XGBoost, eXtreme Gradient Boosting; RF, random forest; ANN, artificial neural network; ROS, Random Over-Sampling; SMOTE, Synthetic Minority Over-sampling Technique. Bold values indicate that the model’s results represent the optimal outcomes within their respective categories of missing value imputation methods and resampling techniques.

### Development and assessment of the best-performing model

The LightGBM model utilizing RF imputation emerged as the best-performing model for early warning of DILI in older patients. The model underwent 5-fold CV on the training set using the same hyperparameters and input variables and was subsequently evaluated on an independent testing set. Receiver operating characteristic (ROC) curve analysis demonstrated outstanding performance, with a mean AUC of 1.0000 (1.0000, 1.0000) for the training set and 0.9829 (0.9737, 0.9904) for the testing set. The results of the 5-fold CV further validated the robustness of the model.

The LightGBM model achieved a mean accuracy of 0.9451 ± 0.0037, indicating a high degree of consistency across the cross-validation folds. Detailed cross-validation accuracy metrics are provided in Supplementary Table S6. The AUC values and mean accuracy were notably consistent between the training and testing sets, demonstrating the model’s robustness and generalizability.

DCA showed that the LightGBM model provided a superior mean net benefit across most threshold probability ranges (Supplementary Figure S2D). With a Brier score of 0.0381 and calibration plots closely aligning with the observed outcomes, the model’s reliability and calibration were further affirmed.

### SHAP and importance score of variables

To provide an intuitive interpretation of the LightGBM model, we leveraged the SHAP algorithm and variable importance scores to gain insights into the contributions of different variables toward DILI warning. The results of the SHAP value analysis and the importance score ranking were consistent, identifying 200 variables as significant contributors to DILI warning (Supplementary Table S7).

**FIGURE 2:**
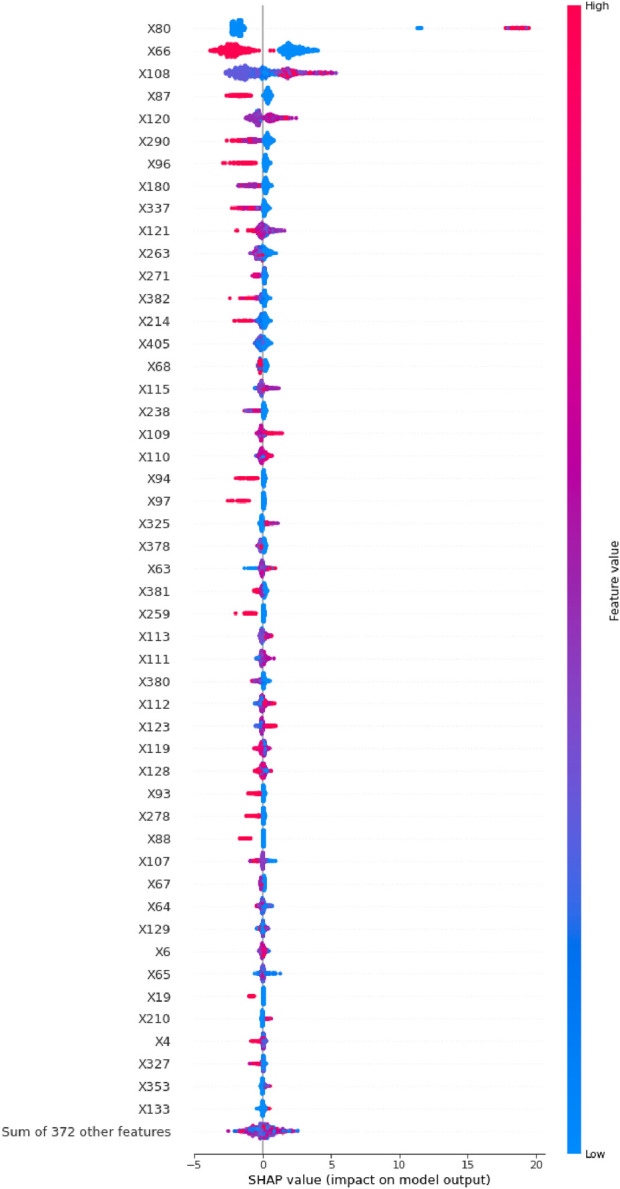
The SHAP beeswarm plot of the top 50 most important variables in the LightGBM model with RF imputation.

**FIGURE 3:**
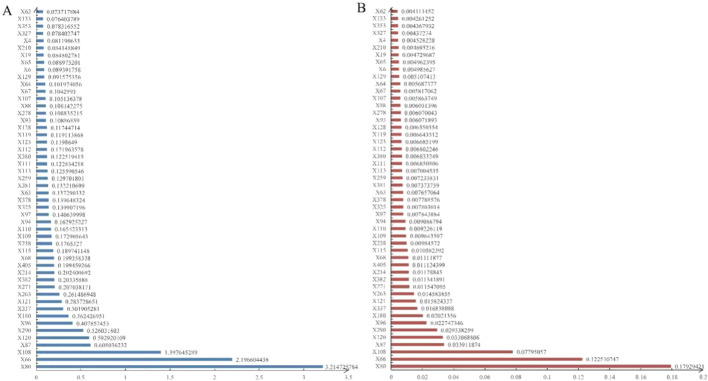
The mean SHAP values **(A)** and variable importance ranking **(B)** in the LightGBM model using RF imputation.

To visualize these findings, a SHAP beeswarm plot (Figure 2) was generated, illustrating the directional impacts of the top 50 most influential variables in the testing set. Additionally, a SHAP bar plot (Figure 3A) ranked these top 50 variables based on their importance. Key contributing factors included the timing of DILI relative to surgery, whether surgery was performed, and the maximum slopes of ALT, AST, ALP, GGT, ALB, LDL-C, HDL-C, TBil, pro-brain natriuretic peptide (Pro-BNP), total bile acids (TBA), and CHO. Additional significant variables included HBeAg semi-quantitative levels, the administration of liver-protecting medications upon admission, use of diuretics, antibiotics, narcotic analgesics, and the presence of liver or gallbladder diseases or cancers.

The variable importance scores, presented in Figure 3B, corroborated the rankings observed in the SHAP bar plot, further validating the robustness of these key predictors.

**FIGURE 4:**
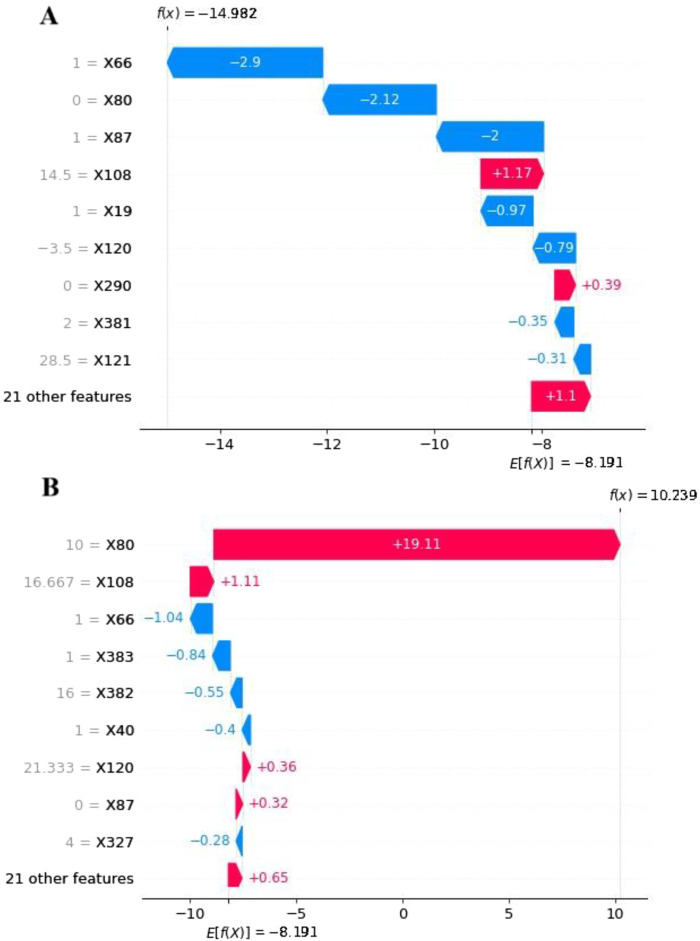
The SHAP waterfall plots for 2 patients: **(A)** Without DILI and **(B)** With DILI Using the LightGBM Model with RF Imputation. The E [f(x)] represents the average warning value of the model without any feature input. The arrows indicate the contributions of each feature, pointing towards the direction of the warning result, with their length representing its importance. The f(x) denotes the actual forecast value of the model for specific individuals.

To explore feature contributions at the individual patient level, we analyzed two randomly selected cases from the testing set using SHAP waterfall plots (Figure 4). Both scenarios—with and without resampling—were considered. In the case predicting a negative outcome, the primary influencing factors included surgery (−2.9%), the timing of DILI relative to surgery (−2.12%), and liver cancer or metastatic liver cancer (2%), resulting in a forecast value (f(x)) of −14.982 (< E [f(x)]). Conversely, for a case predicting a positive outcome, the major contributors were the timing of DILI relative to surgery (19.11%), the maximum slope of ALT (1.11%), and surgery (−1.04%), yielding a forecast value (f(x)) of 10.239 (> E [f(x)]). As variable names could not be annotated directly in Figures 2–4, the specific names of the variables should be referenced in Supplementary Table S3.

## Discussion

DILI represents a significant ADR among older patients, where delayed detection and inadequate management can markedly increase the risk of acute liver failure and fatal complications (Reuben et al., 2010). Initial manifestations of DILI often involve abnormal elevations in liver enzymes or bile acids. Early identification of risk factors in this vulnerable population is crucial for enabling timely interventions, thereby improving patient outcomes and quality of life.

Traditionally, scholars have relied primarily on monitoring abnormalities in liver enzyme or bilirubin levels to detect DILI (Kong et al., 2021). However, such biomarkers can also be influenced by other diseases or surgical procedures, reducing the specificity and efficiency of detection systems based solely on these indicators (Kong et al., 2021). Although prior studies have applied ML techniques to detect ADRs, including cardiovascular events caused by analgesics or concurrent adverse reactions (Liu et al., 2018; Bagattini et al., 2019), there remains a notable gap in literature regarding the development of early warning models specifically for DILI.

In the present study, we successfully developed and validated multiple ML models to warn of DILI using routine clinical, pharmacological, and laboratory data. Among the models, LightGBM demonstrated exceptional performance, achieving an AUC of 0.9829 in the testing set. The minimal difference between training and testing AUCs indicated the model’s strong stability and generalizability. Although resampling techniques such as ROS and SMOTE were applied to address class imbalance, they did not yield significant performance improvements. These findings provide compelling evidence for the further development of tailored DILI warning systems, which could be expanded to ADR warnings more broadly in older populations. An early warning system based on our findings has the potential to enhance physician decision-making, improve ADR detection and intervention rates, and substantially strengthen medication safety, particularly in both tertiary and community healthcare settings.

Using SHAP values and importance score analyses, we further identified key factors contributing to DILI warning. Surgery emerged as a critical risk factor. Surgical-related features—including the surgical procedure itself, the timing of DILI onset relative to surgery, and the type of surgery—were highlighted by negative mean SHAP values, suggesting that postoperative liver enzyme or bilirubin abnormalities should not immediately be attributed to DILI without considering surgical impacts. This was particularly evident following surgeries involving the liver, gallbladder, pancreas, or other digestive organs. Previous studies have demonstrated that surgeries involving these systems can lead to elevated liver enzymes and bilirubin levels due to trauma, hemolysis, or impaired gastrointestinal function (Guo et al., 2016; Sano et al., 2021; He et al., 2023; Soneda et al., 2024). Similarly, brain surgeries, cardiac surgeries, and musculoskeletal surgeries have been associated with postoperative enzyme elevations, while surgeries involving the urinary system or peripheral vasculature appeared less impactful (Kim et al., 2019; Oh et al., 2020; Lott and Landesman, 1984).

Peak slopes of laboratory indicators, particularly ALT, AST, ALP, GGT, ALB, LDL-C, HDL-C, TBil, Pro-BNP, TBA, and CHO, were identified as strong warning factors for DILI. Since all patients exhibited abnormal liver function during hospitalization, neither peak values nor average levels alone were sufficient predictors. Instead, patients who developed sudden dynamic changes in these indicators during hospitalization, particularly those without pre-existing hepatobiliary disease—were more likely to experience DILI.

Interestingly, myocardial markers such as Pro-BNP, Mb, and troponin T were associated with negative mean SHAP values, indicating a potential protective effect against DILI warnings. This finding may reflect the contribution of cardiac injury to elevated AST levels, a marker shared between cardiac muscle, skeletal muscle, and the liver (Panteghini, 1990). Given that a substantial proportion of the cohort had cardiovascular or renal diseases and underwent related surgeries, elevated myocardial enzymes may confound liver enzyme interpretation, necessitating cautious evaluation of AST elevations in the clinical context.

Drug exposures played a major role in the warning model, with 18 out of the top 50 factors being drug related. Notably, hepatoprotective drugs appeared as protective factors, characterized by negative SHAP values. This contrasts with prior predictive models that did not emphasize hepatoprotective therapies (Hu et al., 2020; Hu et al., 2023; Sano et al., 2021; Han et al., 2022; Asai et al., 2023). In our study, hepatoprotective agents were typically administered prophylactically upon admission for patients with pre-existing liver abnormalities, but not immediately for DILI patients until after injury onset. Therefore, the use of hepatoprotective medications before the occurrence of DILI was considered a protective factor in our model.

Other perioperative drugs, such as opioid analgesics and electrolyte modulators, were also classified as risk indicators, likely reflecting surgery complexity and perioperative management rather than direct hepatotoxicity (Xia et al., 2024).

Antibiotics emerged prominently among the DILI-associated drugs. Given their frequent use among older adults for treating infections and preventing postoperative complications (Millett et al., 2013; Hayward et al., 2019; Tuddenham et al., 2022), antibiotic exposure was a substantial contributor to DILI risk. Our findings are consistent with prior reports indicating that up to 64% of DILI cases are attributable to antibiotics (Park et al., 2021). In our study, 72.5% of patients received antibiotics, with a DILI incidence of 14.55%. β-Lactamase inhibitors, carbapenems, and cephalosporins were among the most frequently implicated agents, highlighting the need for judicious antibiotic use in this vulnerable population.

Despite its strengths, this study has several limitations. First, the model was developed using retrospective data and lacked external validation; future prospective cohort studies are warranted to confirm its predictive accuracy and stability. Second, reliance on routine clinical data may limit model robustness. Third, the perfect AUC (1.000) observed in the training set raises concerns about potential overfitting, possibly due to the retrospective design or suboptimal feature selection. Fourth, missing data on hepatitis markers—important for early DILI detection—necessitated imputation strategies, which may have introduced bias. Finally, while the model incorporated dynamic laboratory indicators, it did not account for temporal associations between laboratory changes and drug administration patterns.

Future research should focus on prioritizing clinically relevant features, conducting external validation using independent datasets, and refining the model to better identify high-risk drugs contributing to liver injury. Addressing these gaps will be critical to ensuring the broader applicability and clinical utility of early warning systems for DILI.

## Conclusion

This study successfully developed and validated ML models using routine clinical data to provide early warnings for DILI in older patients. Among the models, the LightGBM model demonstrated superior performance, and its interpretability was enhanced through SHAP analysis. This model can be integrated into hospital information systems to enable automated alerts and tracking of DILI cases, supporting earlier clinical interventions. Our methodological framework not only addresses DILI but also offers a foundation adaptable to detecting other ADRs, such as renal and hematological toxicities, by rapidly identifying drug-related safety issues and aiding clinical decision-making. The proposed ADR early warning system, based on this approach, holds promise for improving ADR detection and intervention rates, thereby enhancing medication safety for older patients across various healthcare settings. Future research should focus on validating these models in larger, multicenter cohorts and incorporating additional data sources, including temporal relationships between drug administration and laboratory results, imaging findings, and detailed clinicopathological information, to further enhance predictive accuracy. Ultimately, integrating such warning models into routine clinical practice could enable real-time decision-making, support personalized patient management, and contribute to improved patient outcomes.

## Data Availability

The raw data supporting the conclusions of this article will be made available by the authors, without undue reservation.
