# Supplementary material for: Development of a warning model for drug-induced liver injury in the older patients
Source: Front Pharmacol. 2025 May 20;16:1603089. doi: 10.3389/fphar.2025.1603089 (PMC12129776; doi:10.3389/fphar.2025.1603089)
Supplement: Supplementary file 1 [file Supplementaryfile1.docx]

**Supplementary Table 1. Characteristics of included patients in training and testing sets**

| **Variables (n [%] or median [IQR])** | **Training set (N=2880)** | **Testing set (N=720)** | ***P*** |
| --- | --- | --- | --- |
| **DILI, n** |  |  |  |
| Yes | 544(18.89%) | 110 (15.28%) | 0.027 |
| No | 2336 (81.11%) | 610 (84.72%) |  |
| **Age, years** | 68.00[65.00,74.00] | 69.00[65.00,75.00] | 0.248 |
| **Gender, n (%)** |  |  |  |
| Male | 1695 (58.85%) | 410 (56.94%) | 0.353 |
| Female | 1185 (41.15%) | 310 (43.06%) |  |
| **Marital status, n (%)** |  |  |  |
| Married | 2641 (91.70%) | 661 (91.81%) | 1.000 |
| Single/divorced/widowed | 239 (8.30%) | 59 (8.19%) |  |
| **Allergic history, n (%)** |  |  |  |
| No | 2642 (91.74%) | 659 (91.53%) | 0.880 |
| Yes | 238 (8.26%) | 61 (8.47%) |  |
| **Ethnic groups, n (%)** |  |  |  |
| Han nationality | 2726 (94.65%) | 676 (93.89%) | 0.412 |
| other nationality | 154 (5.35%) | 44 (6.11%) |  |
| **Smoking history, n (%)** |  |  |  |
| No | 2128 (73.89%) | 535 (74.31%) | 0.849 |
| Yes | 752 (26.11%) | 185 (25.69%) |  |
| **Number of cigarettes among smoking patients, n** | 20.00[10.00, 20.00] | 19.50[10.00, 20.00] | 0.300 |
| **Drinking history, n (%)** |  |  |  |
| No | 2257 (78.37%) | 568 (78.89%) | 0.800 |
| Yes | 623 (24.63%) | 152 (21.11%) |  |
| **Average alcohol consumption among patients with a history of alcohol use, g** | 100.00[100.00,250.00] | 150.00[100.00,250.00] | 0.311 |
| **Surgery, n (%)** |  |  |  |
| No | 1352 (46.94%) | 327 (45.42%) | 0.478 |
| Yes | 1528 (53.06%) | 393 (54.58%) |  |
| **Number of surgeries, n (%)** | | |  |
| 0 | 1352 (46.94%) | 327 (45.42%) | 0.640 |
| 1 | 1482(51.46%) | 377 (52.36%) |  |
| 2 | 43(1.49%) | 15 (2.08%) |  |
| 3 | 3 (0.11%) | 1 (0.14%) |  |
| **Surgical organ, n (%)** |  |  |  |
| Total | 1798 (100.00%) | 449 (100.00%) |  |
| Heart/Aorta | 324 (11.25%) | 98 (13.61%) | 0.855 |
| Brain | 104 (3.61%) | 23 (3.19%) |  |
| Peripheral Vessels | 12 (0.42%) | 3 (0.42%) |  |
| Liver | 458 (15.90%) | 110 (15.28%) |  |
| Biliary, Pancreas or Spleen | 342 (11.87%) | 89 (12.36%) |  |
| Chest (Lungs or Mediastinum) | 95 (3.30%) | 23 (3.19%) |  |
| Digestive System, Abdominal Cavity (Other than Liver, Biliary, Pancreas, Spleen) | 223 (7.74%) | 48 (6.67%) |  |
| Skeleton (Limbs or Spine) | 122 (4.24%) | 29 (4.03%) |  |
| Kidneys and Urinary System | 60 (2.08%) | 12 (1.67%) |  |
| Others Organ | 58 (2.01%) | 14 (1.94%) |  |
| **Medical history in the 10 days prior to this hospitalization, n (%)** | | |  |
| Yes | 1018 (35.35%) | 255 (35.42%) | 1.00 |
| No | 1862 (64.65%) | 465 (64.58%) |  |
| **Number of hospitalizations in this hospital in the past year, n (%)** | | |  |
| 0 | 2195 (76.21%) | 561 (77.92%) | 0.816 |
| 1-5 | 610 (21.18%) | 142 (19.72%) |  |
| 6-10 | 61 (2.12%) | 14 (1.94%) |  |
| 10- | 14 (0.49%) | 3 (0.42%) |  |
| **Level of nursing care upon admission, n (%)** | | |  |
| Specialized nursing care | 142 (4.93%) | 41 (5.69%) | 0.620 |
| Primary care | 1543 (53.58%) | 390 (54.17%) |  |
| Secondary care | 1195 (41.49%) | 289 (40.14%) |  |
| **Type of admission, n (%)** |  |  |  |
| Emergent or Referral | 1238 (42.99%) | 335 (46.53%) | 0.093 |
| Elective | 1642 (57.01%) | 385 (53.47%) |  |
| **Method of admission, n (%)** | | |  |
| On foot | 1750 (60.76%) | 417 (57.92%) | 0.039 |
| Wheel chair | 120 (4.17%) | 20 (2.78%) |  |
| Gurney | 1010 (35.07%) | 283 (39.30%) |  |
| **Viral hepatitis laboratory test results** | | |  |
| HAV-IgG (S/CO) | 1.00[1.00,1.00] | 1.00[1.00,1.00] | 0.931 |
| HBsAb semi-quant (IU/L) | 5.12[2.00,74.17] | 4.98[2.00,72.80] | 0.584 |
| HBsAg semi-quant (COI) | 0.40[0.30,0.47] | 0.40 [0.27,0.47] | 0.628 |
| HBeAb semi-quant (COI) | 1.00[0.97,1.37] | 1.00[0.96,1.38] | 0.746 |
| HBeAg semi-quant (COI) | 0.09[0.06,0.10] | 0.09[0.06,0.10] | 0.697 |
| HBcAb semi-quant (COI) | 0.13[0.01,1.00] | 0.17[0.01,1.00] | 0.508 |
| HP-HBV VL (IU/mL) | 0.00[0.00,0.00] | 0.00[0.00,0.00] | 0.783 |
| anti-HCV (COI) | 0.04[0.03,0.04] | 0.04[0.03,0.04] | 0.550 |
| HP-HCV VL (IU/mL) | 0.00[0.00,0.00] | 0.00[0.00,0.00] | 0.781 |
| HDV-IgM (S/CO) | 0.00[0.00,0.00] | 0.00[0.00,0.00] | 0.452 |
| HEV-IgG (S/CO) | 0.00[0.00,0.00] | 0.00[0.00,0.00] | 0.568 |
| HEV-IgM (S/CO) | 0.00[0.00,0.00] | 0.00[0.00,0.00] | 0.879 |

Abbreviations: HAV-IgG, Hepatitis A Virus Immunoglobulin G; HBsAb semi-quant, Hepatitis B Surface Antibody semi-quantitative; HBsAg semi-quant, Hepatitis B Surface Antigen semi-quantitative; HBeAb semi-quant, Hepatitis B e Antibody semi-quantitative; HBeAg semi-quant, Hepatitis B e Antigen semi-quantitative; HBcAb semi-quant, Hepatitis B Core Antibody semi-quantitative; HP-HBV VL, High Precision Hepatitis B Virus Viral Load; anti-HCV, Antibody to Hepatitis C Virus; HP-HCV VL, High Precision Hepatitis C Virus Viral Load; HDV-IgM, Hepatitis D Virus Immunoglobulin M; HEV-IgG, Hepatitis E Virus Immunoglobulin G; HEV-IgM, Hepatitis E Virus Immunoglobulin M. Note, For categorical variables, frequencies and percentages are reported. For continuous variables, data are presented as median and interquartile range [IQR] for non-normally distributed data. P values < 0.05 were considered statistically significant.

**Supplementary Table 2. Drugs associated with DILI and incidence rates**

| **Classification of drugs** | **Number of patients with DILI, n** | **Number of patients on medication, n** | **Incidence rate (%)** |
| --- | --- | --- | --- |
| Coagulants | 3 | 1210 | 0.25% |
| Gastrointestinal motility agents, antiemetics, and emetics | 25 | 1833 | 1.36% |
| Lipid-regulating agents and anti-atherosclerotic drugs | 26 | 725 | 3.59% |
| Calcium channel blockers | 2 | 1258 | 0.16% |
| Antipyretic analgesics and anti-inflammatory drugs | 65 | 1549 | 4.20% |
| Antiviral drugs | 10 | 317 | 3.15% |
| Antiepileptic drugs | 23 | 229 | 10.04% |
| Antihypertensive drugs | 2 | 1022 | 0.20% |
| Anxiolytics | 5 | 601 | 0.83% |
| Antituberculosis drugs | 6 | 41 | 14.63% |
| Antipsychotic drugs | 7 | 174 | 4.02% |
| Antimicrobial agents | 380 | 2611 | 14.55% |
| Anticoagulants | 136 | 2105 | 6.46% |
| Antianemic drugs | 4 | 376 | 1.06% |
| Antigout drugs | 2 | 109 | 1.83% |
| Antiarrhythmic drugs | 2 | 215 | 0.93% |
| Antiplatelet agents | 2 | 616 | 0.32% |
| Anxiolytic agents | 10 | 65 | 15.38% |
| Antifungal drugs | 37 | 300 | 12.33% |
| Antineoplastic drugs | 39 | 584 | 6.68% |
| Anesthetics and adjunctive medications | 15 | 2555 | 0.59% |
| Urological agents | 4 | 1871 | 0.21% |
| Immunostimulants | 5 | 281 | 1.78% |
| Sympathomimetics and sympatholytics | 1 | 1088 | 0.09% |
| Bronchodilators | 2 | 495 | 0.40% |
| Expectorants | 5 | 1576 | 0.32% |
| Adrenal corticosteroids and adrenocorticotropic hormones | 12 | 1317 | 0.91% |
| Biological products | 2 | 390 | 0.51% |
| Insulin and other glucose-lowering agents | 2 | 682 | 0.29% |
| Nutritional agents | 9 | 1181 | 0.76% |
| Drugs affecting bone metabolism | 1 | 48 | 2.08% |
| Diagnostic agents | 2 | 2199 | 0.09% |
| Cough suppressants | 2 | 229 | 0.87% |
| Sedatives, hypnotics, and anticonvulsants | 10 | 1772 | 0.56% |
| Analgesics | 19 | 2230 | 0.85% |
| Drugs for chronic heart failure | 1 | 366 | 0.27% |
| Drugs for peptic ulcer disease and gastroesophageal reflux disease | 54 | 2023 | 2.67% |
| Traditional Chinese medicine | 6 | 780 | 0.77 |

**Supplementary Table 3. List of variables**

| Number | Variable | Assignment |
| --- | --- | --- |
|  | Drug-Induced Liver Injury | Yes=1; No=0 |
| X1 | Ethnic groups | Han nationality =1; other nationality =0 |
| X2 | Marital status | Married =1; Single/divorced/widowed =0 |
| X3 | Gender | Male =1; Female =0 |
| X4 | Age (years) | Specific values |
| X5 | Height (cm) | Specific values |
| X6 | Weight (kg) | Specific values |
| X7 | Allergic history | Yes =1; No= 0 |
| X8 | Smoking | Yes =1; No= 0 |
| X9 | Number of cigarettes (n/day) | Specific values |
| X10 | Smoking Cessation | Quit smoking = 0, Not quit smoking = 1 |
| X11 | Duration of Smoking (years) | Specific values |
| X12 | Drinking | Yes =1; No= 0 |
| X13 | Average alcohol consumption (g/day) | Specific values |
| X14 | Duration of Alcohol Consumption (year) | Specific values |
| X15 | Alcohol Cessation | Quit drinking = 0, Not quit drinking = 1 |
| X16-X61 | Admission Ward | Laminar Flow Research Ward (X16), Infectious Disease Medical Unit (X17), Trauma Medicine Center Medical Unit (X18), Biliary Surgery Ward (X19), Ear, Nose, Throat - Head and Neck Surgery Medical Unit (X20), Lung Cancer Center Medical Unit (X21), Rheumatology and Immunology Medical Unit (X22), Abdominal Tumor Ward (X23), Hepatic Surgery Ward (X24), Liver Transplant Center (X25), Infectious Disease Medical Unit (X26), Orthopedic Medical Unit (X27), Respiratory and Critical Care Medicine Department (X28), Thyroid Surgery Ward (X29), Tuberculosis Medical Unit (X30), Colorectal Tumor Center Medical Unit (X31), Rehabilitation Medicine Department (X32), Geriatric Medicine Center / Cadre Medical Unit (X33), Urology Medical Unit (X34), Endocrinology and Metabolism Medical Unit (X35), Dermatology and Venereology Medical Unit (X36), General (Special Needs) Ward (X37), Breast Disease Center Medical Unit (X38), Neurology Medical Unit (X39), Neurosurgery Medical Unit (X40), Nephrology Medical Unit (X41), Biological Therapy Research Ward (X42), Pain Medicine Medical Unit (X43), Head and Neck Tumor Ward (X44), Gastric Cancer Center Medical Unit (X45), Gastrointestinal Surgery Ward (X46), Gastroenterology Medical Unit (X47), Mental Health Center Medical Unit (X48), Cardiothoracic Surgery Medical Unit (X49), Cardiology Medical Unit (X50), Thoracic Tumor Ward (X51), Thoracic Surgery Medical Unit (X52), Vascular Surgery Ward (X53), Hematology Medical Unit (X54), Ophthalmology Medical Unit (X55), Pancreatic Surgery Ward (X56), Pancreatitis Center Medical Unit (X57), Plastic Surgery / Burn Unit Medical Unit (X58), Integrated Traditional and Western Medicine Internal Medicine Unit (X59), Oncology Day Care Medical Unit (X60), Intensive Care Medicine Department (X61) |
| X62 | Systolic Blood Pressure | Specific values |
| X63 | Diastolic Blood Pressure | Specific values |
| X64 | Blood glucose | Specific values |
| X65 | HbA1c | Specific values |
| X66 | Surgery | Yes =1; No= 0 |
| X67 | Type of surgery | Emergency surgery=2, elective surgery=1, no surgery = 0 |
| X68 | Surgical Category | No Surgery = 0; Interventional Surgery = 1; Cavity Surgery = 2; Superficial Tissue Surgery = 3; Deep Tissue Surgery = 4; Organ Surgery = 5 |
| X69 | Number of Surgeries During This Hospitalization | Specific values |
| X70-X79 | Involved Organs in Surgery | Heart/Aorta (X70), Brain (X71), Peripheral Vessels (X72), Liver, Biliary, Pancreas or Spleen (X73), Chest (Lungs or Mediastinum) (X74), Digestive System (X75), Abdominal Cavity (Other than Liver, Biliary, Pancreas, Spleen) (X76), Skeleton (Limbs or Spine) (X77), Kidneys and Urinary System (X78), Others Organ (X79) |
| X80 | Relationship Between the Timing of DILIs and Surgery | Specific values (Preoperative DILI = Negative, Postoperative DILI = Positive, No Surgery or No DILI = 0) |
| X81 | Whether treated at an external hospital within 10 days before admission | Yes =1; No= 0 |
| X82 | Number of Hospital Admissions in the Past Year | Specific values |
| X83 | Level of Nursing Care During Admission | Specialized nursing care=0; Primary care=1; Secondary care=2 |
| X84 | Type of admission | Emergent or Referral=2; Elective=1 |
| X85 | Method of admission | On foot=1; Wheel chair=2; Gurney=3 |
| X86-X106 | Disease | Malignant tumors (excluding liver, pancreas, gallbladder) (X86), Liver cancer or liver metastatic cancer (X87), Pancreatic cancer or pancreatic metastatic cancer (X88), Gallbladder cancer or gallbladder metastatic cancer (X89), Bone tumors or metastatic tumors (X90), Viral liver disease (X91), Alcoholic liver disease (X92), Liver cirrhosis (X93), Other liver diseases (including unexplained liver failure) (X94), Acute cerebral infarction or intracranial hemorrhage (excluding cancer) (X95), Gallbladder diseases (excluding cancer) (X96), Pancreatic diseases (excluding cancer) (X97), AECOPD (Acute Exacerbation of Chronic Obstructive Pulmonary Disease) or respiratory failure (X98), Chronic renal insufficiency or renal failure (X99), Bone diseases (excluding cancer) associated with elevated alkaline phosphatase (X100), Acute coronary syndrome or heart disease or heart failure (acute episode) or cardiac arrest (X101), Heart valve disease (moderate/severe) (X102), Venous thromboembolism (X103), Shock (X104), Aortic dissection or aneurysm (X105), Peritonitis/abdominal infection (X106) |
| X107- X123 | The maximum slope of important laboratory indicators related to DILI (Specific values) | Slope of proBNP (X107), ALT (X108), LDL-C (X109), TBA (X110), TBil (X111), TG (X112), ALB (X113), DBil (X114), ALP (X115), Mb (X116), CK-MB (X117), cTnT (X118), CHO (X119), GGT(X120), AST (X121), IBil (X122), HDL-C (X123) |
| X124-X142 | Laboratory indicators that may lead to abnormal liver function (Specific values) | HBsAg quant (X124), HDV-IgM (X125), anti-HCV (X126), HBeAb semi-quant (X127), HBeAg semi-quant (X128), HBcAb semi-quant (X129), HBsAb semi-quant (X130), HEV-IgM (X131), HAV-IgM (X132), HBsAg semi-quant (X133), HP-HBV VL (X134), HBV DNA RT-PCR (X135), AFP (X136), HBsAg Quantitative Test (1:900 Dilution) (X137), HBsAg Quantitative Test (Original Concentration) (X138), HEV-IgG (X139), HAV-IgG (X140), HP-HCV VL (X141), HDV-IgG (X142) |
| X143-X421 | Drug (Specific values) | 5-Hydroxytryptamine receptor antiemetic drugs (X143), 5α-reductase inhibitor drugs for treating benign prostatic hyperplasia (X144), ACEI selective calcium channel blocker drugs (X145), ACEI class antihypertensive drugs (X146), ARB diuretic class antihypertensive drugs (X147), ARB selective calcium channel blocker drugs (X148), ARB class antihypertensive drugs (X149), DDP-4 biguanide class hypoglycemic drugs (X150), DDP-4 inhibitor class hypoglycemic drugs (X151), GLP-1 inhibitor class hypoglycemic drugs (X152), H2 receptor antagonist class gastric acid secretion inhibitors (X153), MRI contrast agents (X154), M receptor antagonist class gastrointestinal antispasmodic drugs (X155), M cholinergic receptor antagonist class bronchodilators (X156), NMDA receptor antagonist class drugs for Alzheimer’s disease and brain metabolism improvement (X157), SGLT-2 inhibitor class hypoglycemic drugs (X158), α receptor antagonist class drugs for treating benign prostatic hyperplasia (X159), α receptor antagonists (X160), α-glucosidase inhibitor class hypoglycemic drugs (X161), β-lactamase inhibitors and their combination formulations with β-lactam antibiotics (X162), β-adrenergic agonist class bronchodilators (X163), γ-aminobutyric acid modulators class antiepileptic drugs (X164), Butyrophenone class antipsychotic drugs (X165), Tricyclic antidepressants (X166), Propionic acid class antipyretic analgesic anti-inflammatory drugs (X167), Propionic acid class antipyretic analgesic anti-inflammatory drugs - local (X168), Intermediate-acting insulin (X169), Central DA receptor agonist class drugs for Parkinson’s disease (X170), Central cough suppressants (X171), Acetic acid class antipyretic analgesic anti-inflammatory drugs (X172), Acetic acid class antipyretic analgesic anti-inflammatory drugs - local (X173), Traditional Chinese medicine class immunosuppressive drugs (X174), Traditional drugs promoting leukocyte proliferation (X175), In vitro diagnostic reagents (X176), Hemostatic drugs acting on blood vessels (X177), Uric acid excretion promoting class anti-gout drugs (X178), Fibrinolytic drugs (X179), Cell regeneration liver-protecting drugs (X180), Gastrointestinal motility promoting drugs (X181), Insulin secretion promoting class hypoglycemic drugs (X182), Brain metabolism promoting class central nervous system stimulants (X183), Drugs promoting coagulation system function (X184), Drugs promoting platelet production (X185), Catechol-O-methyltransferase inhibitor class drugs for Parkinson’s disease (X186), Immune enhancers (X187), Other coagulation promoting drugs (X188), Other anticoagulant drugs (X189), Other anti-Parkinson’s disease drugs (X190), Other anti-angina drugs (X191), Other antiepileptic drugs (X192), Other antitumor drugs and auxiliary treatment drugs (X193), Other antibacterial antibiotics (X194), Other anti-anemia drugs (X195), Other drugs for treating peptic ulcers (X196), Other gastrointestinal drugs (X197), Other gastrointestinal drugs - local (X198), Other dermatological drugs - local (X199), Other ophthalmological drugs (X200), Other ophthalmological drugs - local (X201), Other local anesthetics - local (X202), Other antidepressants (X203), Other anti-Alzheimer’s and brain metabolism improvement drugs (X204), Other cerebrovascular drugs (X205), Other antihypertensive drugs (X206), Other antiemetic drugs (X207), Other parenteral nutrition drugs (X208), Other gastrointestinal antispasmodic drugs (X209), Other sedative-hypnotic anticonvulsants (X210), Other antihypertensive drugs (X211), Other detoxifying drugs (X212), Other antipyretic analgesic anti-inflammatory drugs (X213), Other drugs regulating water, electrolytes, or acid-base balance (X214), Internal medicine nourishing blood and activating blood circulation agents (X215), Internal medicine drugs for promoting blood circulation and removing stasis (X216), Internal medicine drugs for rescuing Yang and restoring the pulse (X217), Internal medicine drugs for calming the liver and extinguishing wind (X218), Internal medicine hemostatic agents (X219), Internal medicine Qi and blood tonics (X220), Internal medicine drugs for activating blood and resolving swelling (X221), Internal medicine drugs for moistening the intestines and relieving constipation (X222), Internal medicine drugs for clearing heat and draining fire (X223), Internal medicine drugs for clearing heat and detoxifying (X224), Internal medicine drugs for clearing heat and promoting urination (X225), Internal medicine drugs for soothing the liver and stomach (X226), Internal medicine Qi-tonifying and Yin-nourishing agents (X227), Internal medicine Qi-tonifying and pulse-restoring agents (X228), Internal medicine Qi-tonifying and activating blood agents (X229), Internal medicine Qi-moving and blood-activating agents (X230), Internal medicine Qi tonics (X231), Internal medicine kidney-tonifying and calming agents (X232), Internal medicine warm and acrid exterior-releasing agents (X233), Internal medicine dual Qi and Yin tonics (X234), Coagulation factor preparations (X235), Coagulation factor preparations - local (X236), Diuretic class antihypertensive drugs (X237), Liver-protecting and gallbladder drugs (X238), Digestive aids (X239), Monoamine oxidase-B inhibitor class drugs for Parkinson’s disease (X240), Norepinephrine and specific serotoninergic antidepressants (X241), Biguanide class hypoglycemic drugs (X242), Folic acid class anti-anemia drugs (X243), Morphine class poisoning detoxifying drugs (X244), Pyrrolidone class brain metabolism activators (X245), Pyrazalone class antipyretic analgesic anti-inflammatory drugs (X246), Phenothiazine class antipsychotic drugs (X247), Inhalation general anesthetics (X248), Peripheral vascular dilators class antihypertensive drugs (X249), Azole class antifungal drugs (X250), Quinolone class antibacterial drugs (X251), Oxazolidinone class antibacterial drugs (X252), Organ function tests and other diagnostic agents (X253), Thiazolidinedione class biguanide hypoglycemic drugs (X254), Thiazolidinedione class hypoglycemic drugs (X255), Tetracycline class antibiotics (X256), Pituitary posterior lobe uterine contraction drugs (X257), Pituitary hormones and related drugs (X258), Basic metabolism class liver and gallbladder disease drugs (X259), Compound chemically synthesized antibacterial drugs (X260), Compound bronchodilators (X261), Compound anti-Parkinson’s disease drugs (X262), Compound electrolyte infusion and dialysis solutions (X263), Peripheral cough suppressants (X264), Surgical heat-clearing and detoxifying agents (X265), Surgical heat-clearing and detoxifying agents - local (X266), Surgical warming and Qi-moving blood-activating dispersing agents - local (X267), Polyclonal or monoclonal antibody class immunosuppressive drugs (X268), Polyene class antifungal drugs (X269), Macrolide class antibiotics (X270), Cephalosporin class antibiotics (X271), Bulk-forming laxatives (X272), Dry eye disease medications - local (X273), Broad-spectrum antiviral drugs (X274), Broad-spectrum anthelmintic and insecticidal drugs (X275), Drugs prolonging action potential duration class antiarrhythmic drugs (X276), Cardiac glycoside class drugs (X277), Drugs affecting cholesterol synthesis (X278), Drugs affecting cholesterol absorption and transport (X279), Drugs affecting lipoprotein transport and breakdown (X280), Uric acid production inhibitors class anti-gout drugs (X281), Granulocyte infiltration inhibitors class anti-gout drugs (X282), Fibrinolytic system inhibitors (X283), Bone resorption inhibitors (X284), Antimetabolite class antitumor drugs (X285), Antiproliferative drugs (X286), Anti-infective dermatological drugs - local (X287), Antiflu virus drugs (X288), Antitrichomonal drugs (X289), Anti-inflammatory liver-protecting drugs (X290), Antithyroid drugs (X291), Antiviral drugs for herpes (X292), Antiviral drugs for herpes - local (X293), Leukotriene receptor antagonists class bronchodilators (X294), Antihistamines (X295), Antiviral drugs for hepatitis (X296), Antitumor traditional Chinese medicine (X297), Antitumor antibiotics (X298), Antitumor hormone class drugs (X299), Antitumor targeted drugs (X300), Anticholinergic class anti-Parkinson’s disease drugs (X301), Anticholinesterase class cholinergic drugs (X302), Antiplatelet drugs (X303), Antiretroviral drugs (X304), Antacids (X305), Adrenergic agonists (X306), M cholinergic receptor antagonists class anticholinergic drugs (X307), Drugs controlling malaria symptoms (X308), Bronchial contrast agents (X309), Drugs improving microcirculation and reducing blood viscosity class cerebrovascular drugs (X310), New structural antipsychotic drugs (X311), Ibuprofen class antipyretic analgesic anti-inflammatory drugs (X312), Xikang class antipyretic analgesic anti-inflammatory drugs (X313), Organophosphate poisoning detoxifying drugs (X314), Lincomycin class antibiotics (X315), Citrate class anticoagulants (X316), Echinocandin class antifungal drugs (X317), Plant-derived antitumor drugs and their derivatives (X318), Antidiarrheal drugs (X319), Aminoglycoside class antibiotics (X320), Amino acid class parenteral nutrition drugs (X321), Cyanide poisoning detoxifying drugs (X322), Drugs for treating diabetes insipidus (X323), Disinfecting, antiseptic, and astringent drugs - local (X324), Lubricant laxatives - local (X325), Lymphatic contrast agents (X326), Osmotic laxatives (X327), Alkylating agents (X328), Ring-pyrrolidone class sedative-hypnotic anticonvulsants (X329), Biological products class promoting leukocyte proliferation (X330), Biological products for treatment (X331), Biological products for treatment - local (X332), Biological products for prevention (X333), Thyroid hormone class drugs (X334), Electrolyte balance regulating drugs (X335), Electrolyte balance regulating drugs - local (X336), Potassium-sparing diuretics and carbonic anhydrase inhibitors (X337), Disease-specific enteral nutrition drugs (X338), Skin cleansers - local (X339), Probiotics (X340), Direct factor IIa inhibitors (X341), Direct Xa inhibitors (X342), Direct vasodilators acting on small blood vessels class peripheral vasodilators (X343), Direct vasodilators acting on small blood vessels class peripheral vasodilators - local (X344), Short-acting insulin (X345), Nitroimidazole antibiotics (X346), Nitrate esters for the treatment of angina (X347), Thiazole antipsychotic drugs (X348), Carbapenems and other β-lactam antibiotics (X349), Sulfonamide antibiotics - local (X350), Sulfonylurea hypoglycemic agents (X351), Corticosteroids for dermatological use - local (X352), Corticosteroids (X353), Corticosteroids - local (X354), Glycopeptide antibiotics (X355), Tuberculosis bacterium inhibitory drugs (X356), Tuberculosis bacterium killing drugs (X357), Vitamin A or Vitamin D related drugs (X358), Vitamin A or Vitamin D related drugs - local (X359), Vitamin B related drugs (X360), Vitamin C and other vitamins (X361), Vitamin K antagonists’ anticoagulants (X362), Traditional Chinese medicine (X363), Ear, nose, throat, and oral medications - local (X364), Heparin anticoagulants (X365), Adrenergic receptor antagonists’ antihypertensive drugs (X366), Adrenergic corticosteroids for asthma (X367), Adrenergic shock vasopressor drugs (X368), Tumor auxiliary traditional Chinese medicine (X369), Cholinesterase inhibitors for Alzheimer’s disease and brain metabolism improvement (X370), Colloidal bismuth gastric mucosal protectants (X371), Fat emulsion parenteral nutrition drugs (X372), Brain metabolism improvement cerebrovascular drugs (X373), Brain enkephalin inhibitors (X374), Awakening central nervous system stimulants (X375), Benzodiazepine antidotes (X376), Benzodiazepine anxiolytics (X377), Benzodiazepine sedative-hypnotic anticonvulsants (X378), Aniline antipyretic analgesic anti-inflammatory drugs (X379), Plasma and plasma substitutes (X380), Vascular contrast agents and intravascular administration enhancing agents (X381), Loop diuretics (X382), Detoxification and liver protection drugs (X383), Proton pump inhibitors for gastric acid secretion suppression (X384), Ultrasound contrast agents (X385), Ultra-short-acting insulin (X386), Allergic mediator release inhibitors for asthma (X387), Selective serotonin reuptake inhibitors antidepressants (X388), Selective serotonin and norepinephrine reuptake inhibitors antidepressants (X389), Selective calcium channel blockers (X390), General enteral nutrition drugs (X391), Ester local anesthetics (X392), Ester local anesthetics - local (X393), Amide local anesthetics (X394), Amide local anesthetics - local (X395), Acid-base balance regulating drugs (X396), Aldehyde sedative-hypnotic anticonvulsants (X397), Aldose reductase inhibitors (X398), Calcium neuroprotein inhibitors (X399), Calcium neuroprotein inhibitors - local (X400), Sodium channel regulating antiepileptic drugs (X401), Sodium channel blocking antiarrhythmic drugs (X402), Iron preparations for anemia (X403), Long-acting insulin (X404), Opioid analgesics (X405), Enzyme-lowering liver protection drugs (X406), Androgens and anabolic steroids (X407), Glaucoma medications - local (X408), Penicillin antibiotics (X409), Intravenous general anesthetics (X410), Non-benzodiazepine anxiolytics (X411), Non-glycoside positive inotropic drugs (X412), Non-opioid analgesics (X413), Premixed insulin (X414), Orthopedic blood-activating and stasis-removing agents (X415), Orthopedic blood-activating and stasis-removing agents - local (X416), Orthopedic kidney-tonifying and bone-strengthening agents (X417), Osteophyte medications (X418), Skeletal muscle relaxants (X419), Xanthine bronchodilators (X420), Mucolytic agents (X421) |

Abbreviations: HbA1c, Hemoglobin A1c; proBNP, N-terminal pro b-type Natriuretic Peptide; ALT, Alanine Aminotransferase; LDL-C, Low-Density Lipoprotein Cholesterol; TBA, Total Bile Acids; TBil, Total Bilirubin; TG, Triglycerides; ALB, Albumin; DBil, Direct Bilirubin; ALP, Alkaline Phosphatase; Mb, Myoglobin; CK-MB, Creatine Kinase-MB; cTnT, Cardiac Troponin T; CHO, Cholesterol; GGT, Gamma-Glutamyl Transferase; AST, Aspartate Aminotransferase; IBil, Indirect Bilirubin; HDL-C, High-Density Lipoprotein Cholesterol; HBsAg quant, Hepatitis B Surface Antigen Quantification; HDV-IgM, Hepatitis D Virus Immunoglobulin M; anti-HCV, Antibody to Hepatitis C Virus; HBeAb semi-quant, Hepatitis B e Antibody Semi-Quantitative Test; HBeAg semi-quant, Hepatitis B e Antigen Semi-Quantitative Test; HBcAb semi-quant, Hepatitis B Core Antibody Semi-Quantitative Test; HBsAb semi-quant, Hepatitis B Surface Antibody Semi-Quantitative Test; HEV-IgM, Hepatitis E Virus Immunoglobulin M; HAV-IgM, Hepatitis A Virus Immunoglobulin M; HBsAg semi-quant, Hepatitis B Surface Antigen Semi-Quantitative Test; HP-HBV VL, Hepatitis B Virus Load; HBV DNA RT-PCR, Hepatitis B Virus DNA Reverse Transcription Polymerase Chain Reaction; AFP, Alpha-Fetoprotein; HBsAg Quantitative Test (1:900 Dilution), Hepatitis B Surface Antigen Quantitative Test (1:900 Dilution) ; HBsAg Quantitative Test (Original Concentration) , Hepatitis B Surface Antigen Quantitative Test (Original Concentration) ; HEV-IgG, Hepatitis E Virus Immunoglobulin G; HAV-IgG, Hepatitis A Virus Immunoglobulin G; HP-HCV VL, Hepatitis C Virus Load; HDV-IgG, Hepatitis D Virus Immunoglobulin G; ACEI, Angiotensin-Converting Enzyme Inhibitor; ARB, Angiotensin II Receptor Blocker; GLP-1, Glucagon-Like Peptide-1; DPP-4, Dipeptidyl Peptidase-4

**Supplementary Table 4. Clinical characteristics of the patients before and after missing value imputation**

| **Variables (median [IQR])** | **Percentage of missing** | **Before Missing Value Treatment** | **After Mean Imputation** | ***P*** | **After Random Forest Imputation** | ***P*** |
| --- | --- | --- | --- | --- | --- | --- |
| Height, cm | 5.17% | 160.00[155.00,167.00] | 160.00[155.00,167.00] | 0.768 | 160.00[155.00,167.00] | 0.733 |
| Weight, kg | 11.61% | 60.00[51.50,65.50] | 59.22[52.50,65.00] | 0.911 | 59.00[52.00,65.00] | 0.491 |
| Number of cigarettes among smoking patients, n | 4.58% | 20.00 [10.00,20.00] | 15[5.00,20.00] | ＜0.001 | 19.42[10.00,20.00] | 0.895 |
| Duration of smoking in years among smoking patients, year | 3.11% | 40.00 [30.00,40.00] | 30.00 [20.00,40.00] | ＜0.001 | 27.44 [20.00,40.00] | 0.314 |
| Average alcohol consumption among drinking patients, g/day | 7.83% | 100.00 [100.00,250.00] | 56.50 [25.51,150.00] | ＜0.001 | 136.34[100.00,200.00] | 0.631 |
| Drinking duration among drinking patients, year | 6.19% | 30.00 [20.00,40.00] | 30.00 [5.57,40.00] | ＜0.001 | 30.00 [25.27,40.00] | 0.247 |
| HbA1c, % | 10.33% | 6.20 [5.80,7.00] | 6.62 [6.62,6.62] | ＜0.001 | 6.11 [5.70,6.48] | 0.145 |
| **Variables (median [IQR])** | **Percentage of missing** | **Before Missing Value Treatment** | | **After Imputation** | |  |
| HAV-IgG (S/CO) | 98.97% | 0.02[0.01,0.08] | | 1.00[1.00, 1.00] | | ＜0.001 |
| HBsAb semi-quant (IU/L) | 23.97% | 23.52[2.00,128.75] | | 5.11[2.00, 73.70] | | ＜0.001 |
| HBsAg semi-quant (COI) | 24.03% | 0.43[0.39,0.50] | | 0.40[0.30, 0.47] | | ＜0.001 |
| HBeAb semi-quant (COI) | 23.97% | 1.18[0.68,1.45] | | 1.00[0.97, 1.37] | | ＜0.001 |
| HBeAg semi-quant (COI) | 23.97% | 0.09[0.08,0.10] | | 0.09[0.06, 0.10] | | ＜0.001 |
| HBcAb semi-quant (COI) | 23.97% | 0.01[0.01,0.75] | | 0.14 [0.01, 1.00] | | ＜0.001 |
| HP-HBV VL (IU/mL) | 96..75% | 0.00[0.00,192.75] | | 0.00[0.00, 0.00] | | ＜0.001 |
| anti-HCV (COI) | 23.89% | 0.04[0.04,0.04] | | 0.04[0.03, 0.04] | | ＜0.001 |
| HP-HCV VL (IU/mL) | 99.17% | 0.00[0.00,940500.00] | | 0.00[0.00, 0.00] | | ＜0.001 |
| HDV-IgM (S/CO) | 99.53% | 0.05[0.03,0.08] | | 0.00[0.00, 0.00] | | ＜0.001 |
| HEV-IgG (S/CO) | 98.94% | 0.68[0.07,8.75] | | 0.00[0.00, 0.00] | | ＜0.001 |
| HEV-IgM (S/CO) | 96.72% | 0.03[0.02,0.13] | | 0.00[0.00, 0.00] | | ＜0.001 |

Abbreviations: HbA1c, Hemoglobin A1c; Note, For continuous variables, data are presented as median and interquartile range [IQR] for non-normally distributed data. P values < 0.05 were considered statistically significant.

**Supplementary Table 5. The best tuned hyperparameters for ML models in this study.**

| **Model** | **Hyperparameters** | |
| --- | --- | --- |
|  | **Mean Imputation** | **RF Imputation** |
| RF - no resampling | max_depth: None,  min_samples_split: 2,  n_estimators: 100 | max_depth: None,  min_samples_split: 2,  n_estimators: 200 |
| XGBoost- no resampling | learning_rate: 0.2,  max_depth: 5,  n_estimators: 200 | learning_rate: 0.2,  max_depth: 5,  n_estimators: 100 |
| AdaBoost- no resampling | learning_rate: 0.1,  n_estimators: 200 | learning_rate: 0.1,  n_estimators: 200 |
| CatBoost- no resampling | depth: 6,  iterations: 200,  learning_rate: 0.1 | depth: 4,  iterations: 200,  learning_rate: 0.1 |
| LightGBM- no resampling | learning_rate: 0.1,  max_depth: -1,  n_estimators: 200 | learning_rate: 0.1,  max_depth: -1,  n_estimators: 200 |
| ANN- no resampling | activation: tanh,  hidden_layer_sizes: (100,),  solver: adam | activation: tanh,  hidden_layer_sizes: (50,),  solver: adam |
| GDBT- no resampling | learning_rate: 0.1,  max_depth: 5,  n_estimators: 100 | learning_rate: 0.1,  max_depth: 5,  n_estimators: 200 |
| TabNet- no resampling | 'gamma': 1.5,  'lambda_sparse': 0.0001,  'n_a': 16, 'n_d': 16, 'n_steps': 3,  'optimizer_fn': <class 'torch.optim.adam.Adam'> | 'gamma': 1.0, '  lambda_sparse': 1e-05,  'n_a': 8, 'n_d': 8, 'n_steps': 3,  'optimizer_fn': <class 'torch.optim.adam.Adam'> |
| RF -ROS | max_depth: None,  min_samples_split: 2,  n_estimators: 100 | max_depth: None,  min_samples_split: 2,  n_estimators: 100 |
| XGBoost -ROS | learning_rate: 0.1,  max_depth: 7,  n_estimators: 700 | learning_rate: 0.1,  max_depth: 7,  n_estimators: 200 |
| AdaBoost -ROS | learning_rate: 0.1,  n_estimators: 200 | learning_rate: 1.0,  n_estimators: 200 |
| CatBoost -ROS | depth: 8,  iterations: 200,  learning_rate: 0.1 | depth: 8,  iterations: 200,  learning_rate: 0.1 |
| LightGBM-ROS | learning_rate: 0.1,  max_depth: -1,  n_estimators: 100 | learning_rate: 0.1,  max_depth: -1,  n_estimators: 200 |
| ANN-ROS | activation: tanh,  hidden_layer_sizes: (50,),  solver: adam | activation: tanh,  hidden_layer_sizes: (100,),  solver: adam |
| GDBT-ROS | learning_rate: 0.1,  max_depth: 7,  n_estimators: 200 | learning_rate: 0.1,  max_depth: 7,  n_estimators: 200 |
| TabNet-ROS | 'gamma': 1.5,  'lambda_sparse': 0.0001,  'n_a': 16, 'n_d': 16, 'n_steps': 3,  'optimizer_fn': <class 'torch.optim.adam.Adam'> | 'gamma': 1.5,  'lambda_sparse': 0.0001,  'n_a': 16, 'n_d': 16, 'n_steps': 5,  'optimizer_fn': <class 'torch.optim.adam.Adam'> |
| RF - SMOTE | max_depth: None,  min_samples_split: 2,  n_estimators: 100 | max_depth: None,  min_samples_split: 2,  n_estimators: 100 |
| XGBoost - SMOTE | learning_rate: 0.2,  max_depth: 7,  n_estimators: 200 | learning_rate: 0.2,  max_depth: 5,  n_estimators: 200 |
| AdaBoost - SMOTE | learning_rate: 1.0,  n_estimators: 200 | learning_rate: 1.0,  n_estimators: 100 |
| CatBoost - SMOTE | depth: 8,  iterations: 200,  learning_rate: 0.1 | depth: 8,  iterations: 200,  learning_rate: 0.1 |
| LightGBM - SMOTE | learning_rate: 0.1,  max_depth: -1,  n_estimators: 200 | learning_rate: 0.1,  max_depth: 20,  n_estimators: 100 |
| ANN - SMOTE | activation: tanh,  hidden_layer_sizes: (50,),  solver: adam | activation: tanh,  hidden_layer_sizes: (100,),  solver: adam |
| GDBT - SMOTE | learning_rate: 0.1,  max_depth: 7,  n_estimators: 200 | learning_rate: 0.1,  max_depth: 7,  n_estimators: 200 |
| TabNet - SMOTE | 'gamma': 1.5,  'lambda_sparse': 1e-05,  'n_a': 16, 'n_d': 16, 'n_steps': 5,  'optimizer_fn': <class 'torch.optim.adam.Adam'> | 'gamma': 1.0,  'lambda_sparse': 1e-05,  'n_a': 8, 'n_d': 8, 'n_steps': 3,  'optimizer_fn': <class 'torch.optim.adam.Adam'> |

Abbreviations: AdaBoost, Adaptive Boosting; CatBoost, Categorical Boosting; GDBT, Gradient Boosting Decision Tree; LightGBM, Light Gradient Boosting Machine; XGBoost, eXtreme Gradient Boosting; RF, Random Forest; ANN, Artificial Neural Network; ROS, Random Over-Sampling; SMOTE, Synthetic Minority Over-sampling Technique


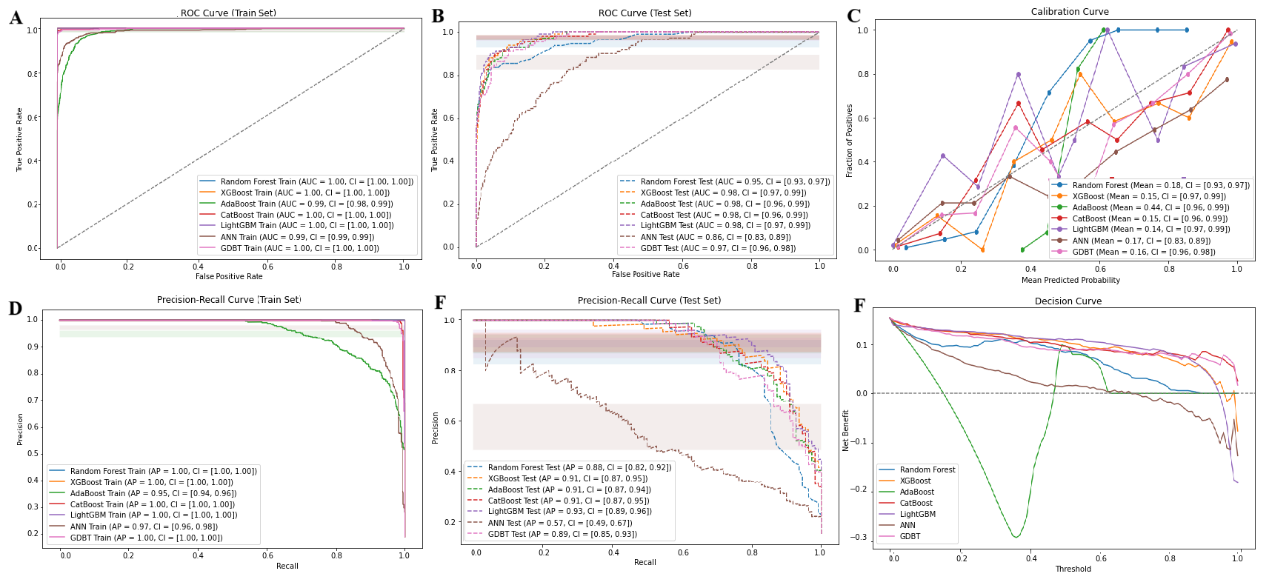


**Supplementary Figure 1. Comprehensive performance of ML on original data using mean imputation.** ROC curves for ML models in the training sets (A) and (B) the testing sets. (C) Calibration curves for ML models in the testing set. PR curve for (D) the training set and (E) the validation set. (F) Decision curve analysis for ML models in the testing set. ML, machine learning.
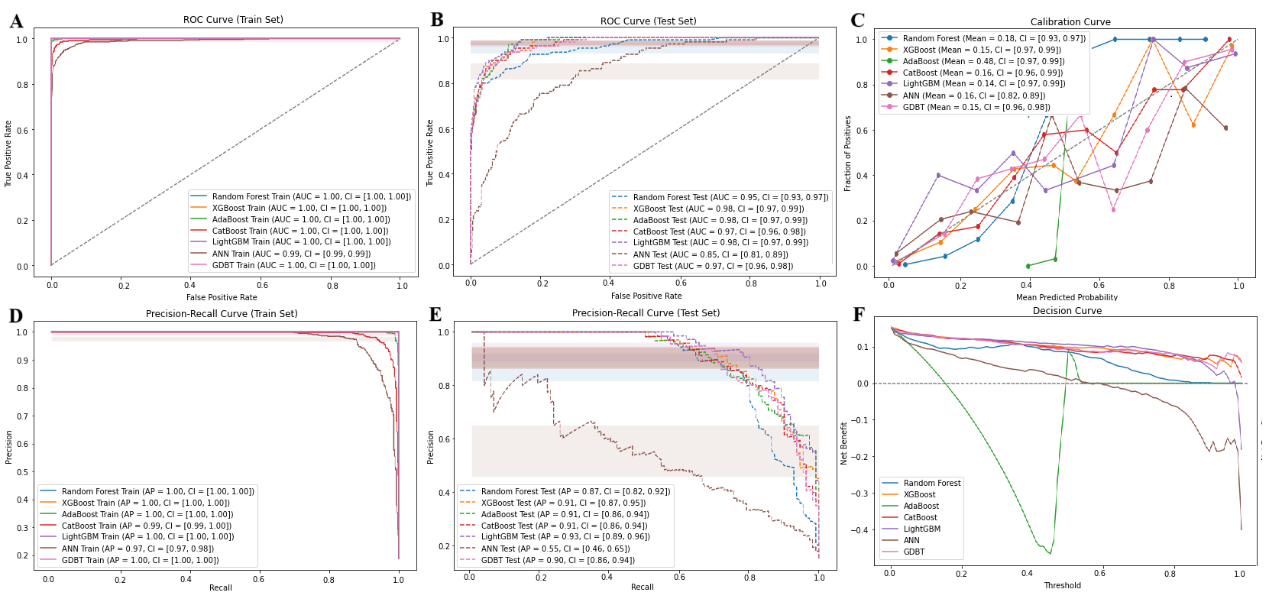


**Supplementary Figure 2. Comprehensive performance of ML on original data using RF imputation.** ROC curves for ML models in the training sets (A) and (B) the testing sets. (C) Calibration curves for ML models in the testing set. PR curve for (D) the training set and (E) the validation set. (F) Decision curve analysis for ML models in the testing set. ML, machine learning; RF, Random Forest


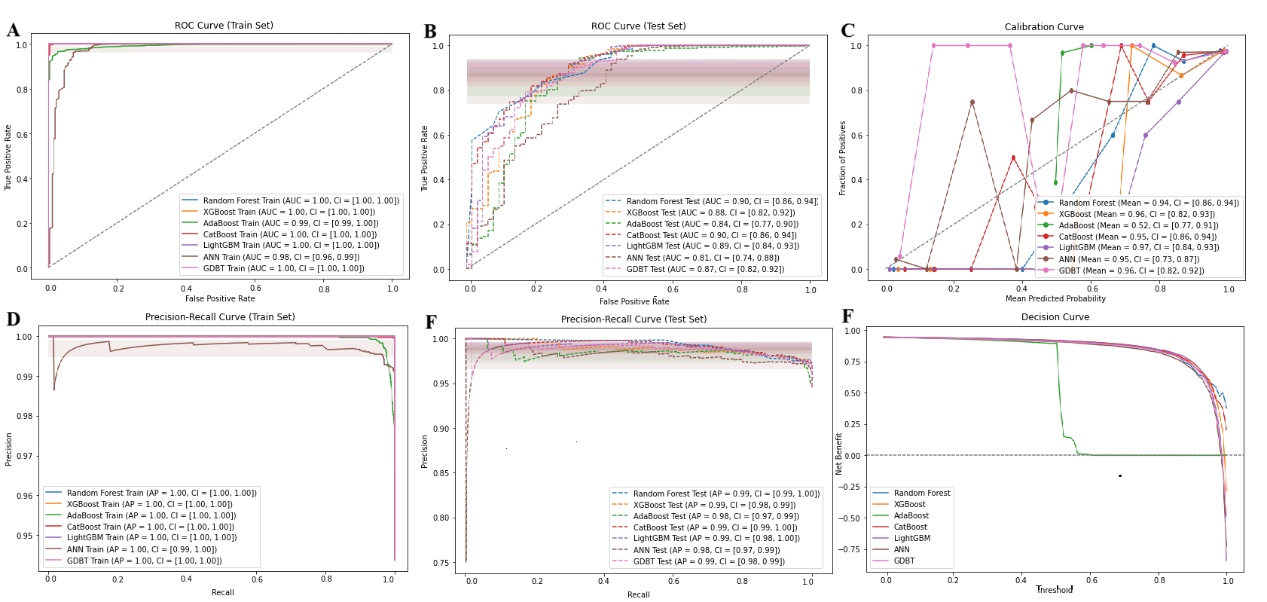


**Supplementary Figure 3. Comprehensive performance of ML using mean imputation on data resampled by ROS.** ROC curves for ML models in the training sets (A) and (B) the testing sets. (C) Calibration curves for ML models in the testing set. PR curve for (D) the training set and (E) the validation set. (F) Decision curve analysis for ML models in the testing set. ML, machine learning; ROS, Random Over-Sampling


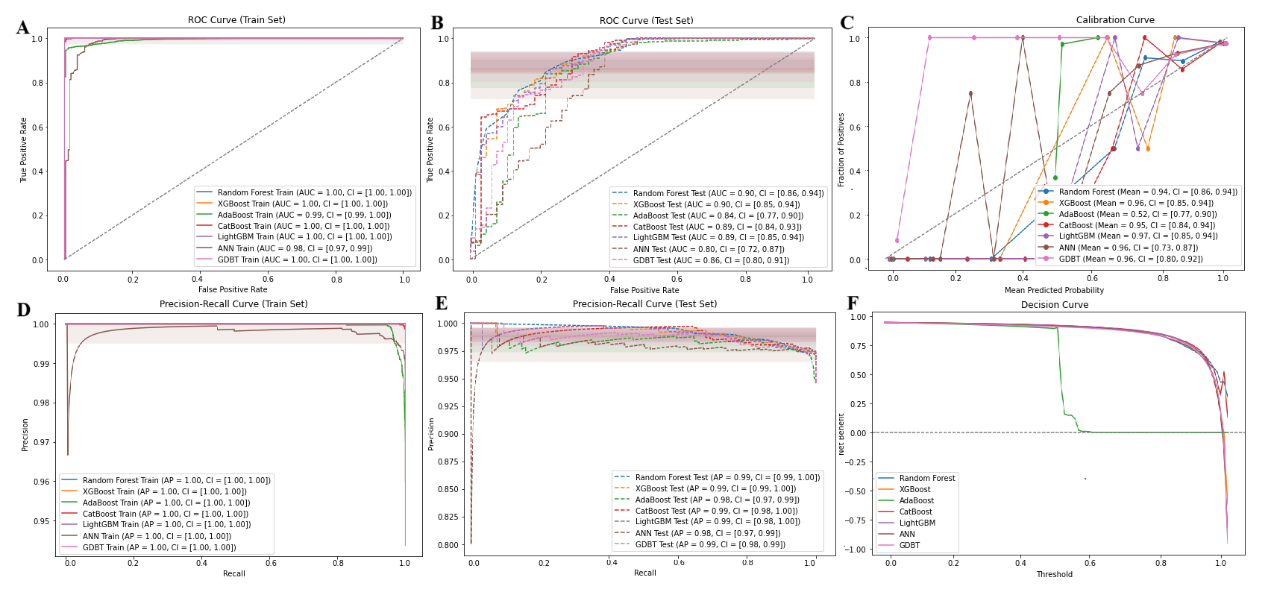


**Supplementary Figure 4. Comprehensive performance of ML using RF imputation on data resampled by ROS.** ROC curves for ML models in the training sets (A) and (B) the testing sets. (C) Calibration curves for ML models in the testing set. PR curve for (D) the training set and (E) the validation set. (F) Decision curve analysis for ML models in the testing set. ML, machine learning; RF, Random Forest; ROS, Random Over-Sampling


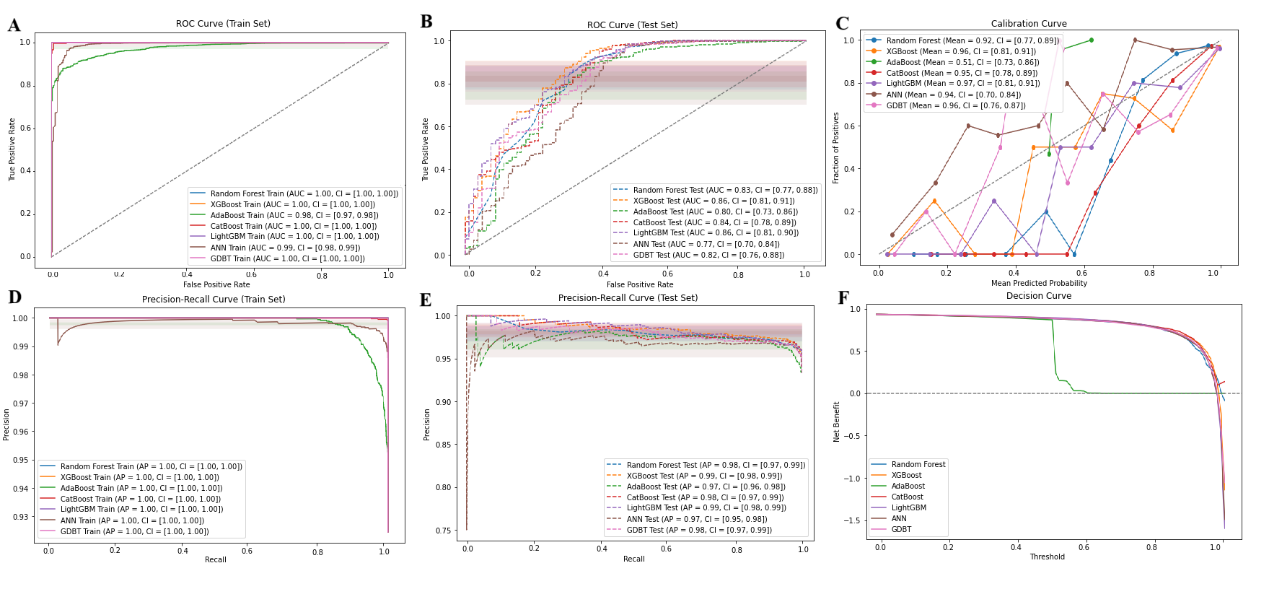
 **Supplementary Figure 5. Comprehensive performance of ML using mean imputation on data resampled by SMOTE.** ROC curves for ML models in the training sets (A) and (B) the testing sets. (C) Calibration curves for ML models in the testing set. PR curve for (D) the training set and (E) the validation set. (F) Decision curve analysis for ML models in the testing set. ML, machine learning; SMOTE, Synthetic Minority Over-sampling Technique


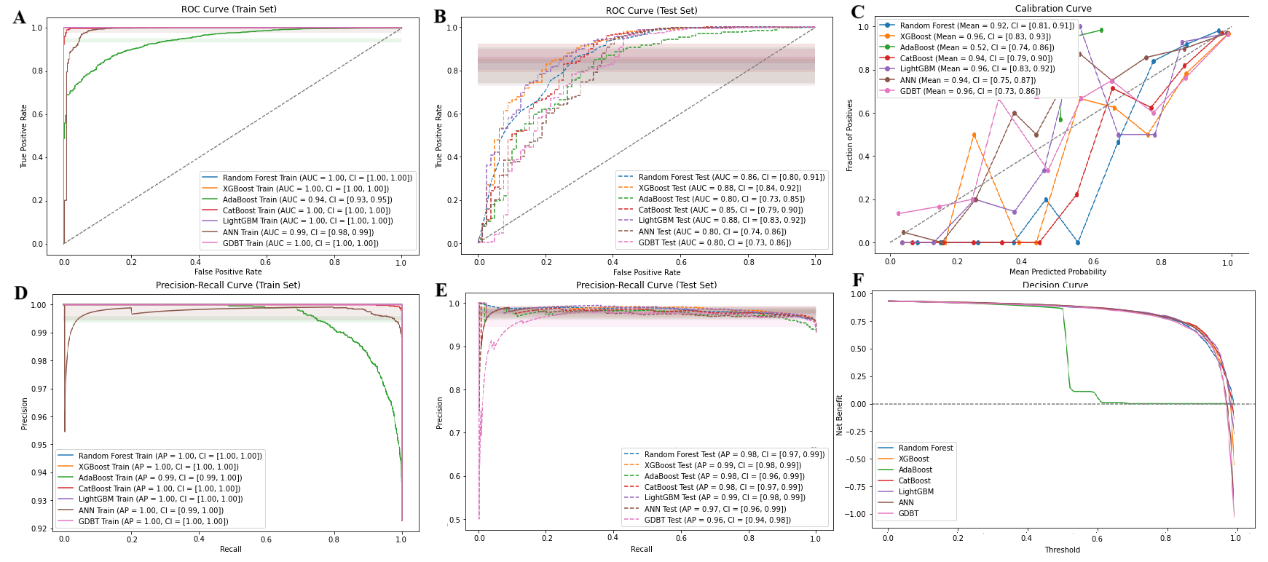


**Supplementary Figure 6. Comprehensive performance of ML using RF imputation on data resampled by SMOTE.** ROC curves for ML models in the training sets (A) and (B) the testing sets. (C) Calibration curves for ML models in the testing set. PR curve for (D) the training set and (E) the validation set. (F) Decision curve analysis for ML models in the testing set. ML, machine learning; RF, Random Forest; SMOTE, Synthetic Minority Over-sampling Technique

**Supplementary Table 6. Results of 5-Fold Cross-Validation in ML**

| **Model** | **Cross-Validation Accuracy** | |
| --- | --- | --- |
|  | **Mean Imputation** | **RF Imputation** |
| RF - no resampling | Fold 1 Accuracy: 0.9028  Fold 2 Accuracy: 0.9028  Fold 3 Accuracy: 0.9167  Fold 4 Accuracy: 0.9219  Fold 5 Accuracy: 0.9201  Mean±SD: 0.9128 ± 0.0084 | Fold 1 Accuracy: 0.9010  Fold 2 Accuracy: 0.9028  Fold 3 Accuracy: 0.9149  Fold 4 Accuracy: 0.9271  Fold 5 Accuracy: 0.9201  Mean±SD: 0.9132 ± 0.0100 |
| XGBoost- no resampling | Fold 1 Accuracy: 0.9358  Fold 2 Accuracy: 0.9427  Fold 3 Accuracy: 0.9462  Fold 4 Accuracy: 0.9444  Fold 5 Accuracy: 0.9479  Mean±SD: 0.9434 ± 0.0042 | Fold 1 Accuracy: 0.9323  Fold 2 Accuracy: 0.9479  Fold 3 Accuracy: 0.9427  Fold 4 Accuracy: 0.9462  Fold 5 Accuracy: 0.9514  Mean±SD: 0.9441 ± 0.0065 |
| AdaBoost- no resampling | Fold 1 Accuracy: 0.9253  Fold 2 Accuracy: 0.9323  Fold 3 Accuracy: 0.9375  Fold 4 Accuracy: 0.9271  Fold 5 Accuracy: 0.9444  Mean±SD: 0.9333 ± 0.0070 | Fold 1 Accuracy: 0.9375  Fold 2 Accuracy: 0.9410  Fold 3 Accuracy: 0.9306  Fold 4 Accuracy: 0.9097  Fold 5 Accuracy: 0.9531  Mean±SD: 0.9344 ± 0.0143 |
| CatBoost- no resampling | Fold 1 Accuracy: 0.9288  Fold 2 Accuracy: 0.9410  Fold 3 Accuracy: 0.9427  Fold 4 Accuracy: 0.9462  Fold 5 Accuracy: 0.9479  Mean±SD: 0.9413 ± 0.0067 | Fold 1 Accuracy: 0.9323  Fold 2 Accuracy: 0.9375  Fold 3 Accuracy: 0.9497  Fold 4 Accuracy: 0.9288  Fold 5 Accuracy: 0.9479  Mean±SD: 0.9392 ± 0.0083 |
| LightGBM- no resampling | Fold 1 Accuracy: 0.9444  Fold 2 Accuracy: 0.9392  Fold 3 Accuracy: 0.9410  Fold 4 Accuracy: 0.9410  Fold 5 Accuracy: 0.9497  Mean±SD: 0.9431 ± 0.0037 | Fold 1 Accuracy: 0.9427  Fold 2 Accuracy: 0.9497  Fold 3 Accuracy: 0.9410  Fold 4 Accuracy: 0.9427  Fold 5 Accuracy: 0.9497  Mean±SD: 0.9451 ± 0.0037 |
| ANN- no resampling | Fold 1 Accuracy: 0.8455  Fold 2 Accuracy: 0.8507  Fold 3 Accuracy: 0.8559  Fold 4 Accuracy: 0.8507  Fold 5 Accuracy: 0.8351  Mean±SD: 0.8476 ± 0.0071 | Fold 1 Accuracy: 0.8646  Fold 2 Accuracy: 0.8628  Fold 3 Accuracy: 0.8507  Fold 4 Accuracy: 0.8420  Fold 5 Accuracy: 0.8212  Mean±SD: 0.8483 ± 0.0159 |
| GDBT- no resampling | Fold 1 Accuracy: 0.9340  Fold 2 Accuracy: 0.9358  Fold 3 Accuracy: 0.9444  Fold 4 Accuracy: 0.9340  Fold 5 Accuracy: 0.9392  Mean±SD: 0.9375 ± 0.0040 | Fold 1 Accuracy: 0.9306  Fold 2 Accuracy: 0.9392  Fold 3 Accuracy: 0.9392  Fold 4 Accuracy: 0.9410  Fold 5 Accuracy: 0.9392  Mean±SD: 0.9378 ± 0.0037 |
| TabNet- no resampling | Fold 1 Accuracy: 0.8514  Fold 2 Accuracy: 0.8792  Fold 3 Accuracy: 0.8778  Fold 4 Accuracy: 0.8847  Fold 5 Accuracy: 0.8653  Mean±SD: 0.8717 ± 0.0120 | Fold 1 Accuracy: 0.9042  Fold 2 Accuracy: 0.9000  Fold 3 Accuracy: 0.9056  Fold 4 Accuracy: 0.9014  Fold 5 Accuracy: 0.9014  Mean±SD: 0.9025 ± 0.0020 |
| RF -ROS | Fold 1 Accuracy: 0.9735  Fold 2 Accuracy: 0.9661  Fold 3 Accuracy: 0.9692  Fold 4 Accuracy: 0.9713  Fold 5 Accuracy: 0.9735  Mean±SD: 0.9707 ± 0.0028 | Fold 1 Accuracy: 0.9735  Fold 2 Accuracy: 0.9661  Fold 3 Accuracy: 0.9692  Fold 4 Accuracy: 0.9713  Fold 5 Accuracy: 0.9735  Mean±SD: 0.9707 ± 0.0028 |
| XGBoost -ROS | Fold 1 Accuracy: 0.9735  Fold 2 Accuracy: 0.9661  Fold 3 Accuracy: 0.9692  Fold 4 Accuracy: 0.9713  Fold 5 Accuracy: 0.9735  Mean±SD: 0.9707 ± 0.0028 | Fold 1 Accuracy: 0.9735  Fold 2 Accuracy: 0.9639  Fold 3 Accuracy: 0.9692  Fold 4 Accuracy: 0.9703  Fold 5 Accuracy: 0.9745  Mean±SD: 0.9703 ± 0.0037 |
| AdaBoost -ROS | Fold 1 Accuracy: 0.9608  Fold 2 Accuracy: 0.9533  Fold 3 Accuracy: 0.9576  Fold 4 Accuracy: 0.9607  Fold 5 Accuracy: 0.9501  Mean±SD: 0.9565 ± 0.0042 | Fold 1 Accuracy: 0.9597  Fold 2 Accuracy: 0.9555  Fold 3 Accuracy: 0.9608  Fold 4 Accuracy: 0.9554  Fold 5 Accuracy: 0.9575  Mean±SD: 0.9578 ± 0.0022 |
| CatBoost -ROS | Fold 1 Accuracy: 0.9714  Fold 2 Accuracy: 0.9650  Fold 3 Accuracy: 0.9692  Fold 4 Accuracy: 0.9703  Fold 5 Accuracy: 0.9713  Mean±SD: 0.9694 ± 0.0024 | Fold 1 Accuracy: 0.9724  Fold 2 Accuracy: 0.9650  Fold 3 Accuracy: 0.9692  Fold 4 Accuracy: 0.9703  Fold 5 Accuracy: 0.9703  Mean±SD: 0.9694 ± 0.0025 |
| LightGBM-ROS | Fold 1 Accuracy: 0.9735  Fold 2 Accuracy: 0.9661  Fold 3 Accuracy: 0.9692  Fold 4 Accuracy: 0.9713  Fold 5 Accuracy: 0.9735  Mean±SD: 0.9707 ± 0.0028 | Fold 1 Accuracy: 0.9735  Fold 2 Accuracy: 0.9650  Fold 3 Accuracy: 0.9692  Fold 4 Accuracy: 0.9713  Fold 5 Accuracy: 0.9745  Mean±SD: 0.9707 ± 0.0034 |
| ANN-ROS | Fold 1 Accuracy: 0.9618  Fold 2 Accuracy: 0.9533  Fold 3 Accuracy: 0.9608  Fold 4 Accuracy: 0.9544  Fold 5 Accuracy: 0.9618  Mean±SD: 0.9584 ± 0.0038 | Fold 1 Accuracy: 0.9661  Fold 2 Accuracy: 0.9639  Fold 3 Accuracy: 0.9692  Fold 4 Accuracy: 0.9597  Fold 5 Accuracy: 0.9671  Mean±SD: 0.9652 ± 0.0033 |
| GDBT-ROS | Fold 1 Accuracy: 0.9703  Fold 2 Accuracy: 0.9597  Fold 3 Accuracy: 0.9692  Fold 4 Accuracy: 0.9703  Fold 5 Accuracy: 0.9713  Mean±SD: 0.9682 ± 0.0043 | Fold 1 Accuracy: 0.9714  Fold 2 Accuracy: 0.9661  Fold 3 Accuracy: 0.9703  Fold 4 Accuracy: 0.9660  Fold 5 Accuracy: 0.9703  Mean±SD: 0.9688 ± 0.0023 |
| TabNet-ROS | Fold 1 Accuracy: 0.9203  Fold 2 Accuracy: 0.9508  Fold 3 Accuracy: 0.9423  Fold 4 Accuracy: 0.9941  Fold 5 Accuracy: 0.9915  Mean±SD: 0.9598 ± 0.0287 | Fold 1 Accuracy: 0.9330  Fold 2 Accuracy: 0.9517  Fold 3 Accuracy: 0.9482  Fold 4 Accuracy: 0.9406  Fold 5 Accuracy: 0.9516  Mean±SD: 0.9450 ± 0.0072 |
| RF - SMOTE | Fold 1 Accuracy: 0.9374  Fold 2 Accuracy: 0.9353  Fold 3 Accuracy: 0.9470  Fold 4 Accuracy: 0.9416  Fold 5 Accuracy: 0.9374  Mean±SD: 0.9397 ± 0.0042 | Fold 1 Accuracy: 0.9427  Fold 2 Accuracy: 0.9321  Fold 3 Accuracy: 0.9427  Fold 4 Accuracy: 0.9384  Fold 5 Accuracy: 0.9374  Mean±SD: 0.9387 ± 0.0039 |
| XGBoost - SMOTE | Fold 1 Accuracy: 0.9449  Fold 2 Accuracy: 0.9417  Fold 3 Accuracy: 0.9544  Fold 4 Accuracy: 0.9512  Fold 5 Accuracy: 0.9512  Mean±SD: 0.9487 ± 0.0047 | Fold 1 Accuracy: 0.9512  Fold 2 Accuracy: 0.9385  Fold 3 Accuracy: 0.9565  Fold 4 Accuracy: 0.9469  Fold 5 Accuracy: 0.9469  Mean±SD: 0.9480 ± 0.0059 |
| AdaBoost - SMOTE | Fold 1 Accuracy: 0.9162  Fold 2 Accuracy: 0.9205  Fold 3 Accuracy: 0.9385  Fold 4 Accuracy: 0.9278  Fold 5 Accuracy: 0.9236  Mean±SD: 0.9253 ± 0.0076 | Fold 1 Accuracy: 0.9279  Fold 2 Accuracy: 0.9215  Fold 3 Accuracy: 0.9343  Fold 4 Accuracy: 0.9289  Fold 5 Accuracy: 0.9236  Mean±SD: 0.9272 ± 0.0044 |
| CatBoost - SMOTE | Fold 1 Accuracy: 0.9406  Fold 2 Accuracy: 0.9343  Fold 3 Accuracy: 0.9459  Fold 4 Accuracy: 0.9395  Fold 5 Accuracy: 0.9459  Mean±SD: 0.9412 ± 0.0044 | Fold 1 Accuracy: 0.9449  Fold 2 Accuracy: 0.9343  Fold 3 Accuracy: 0.9459  Fold 4 Accuracy: 0.9448  Fold 5 Accuracy: 0.9448  Mean±SD: 0.9429 ± 0.0044 |
| LightGBM - SMOTE | Fold 1 Accuracy: 0.9470  Fold 2 Accuracy: 0.9417  Fold 3 Accuracy: 0.9576  Fold 4 Accuracy: 0.9469  Fold 5 Accuracy: 0.9544  Mean±SD: 0.9495 ± 0.0057 | Fold 1 Accuracy: 0.9491  Fold 2 Accuracy: 0.9385  Fold 3 Accuracy: 0.9565  Fold 4 Accuracy: 0.9469  Fold 5 Accuracy: 0.9544  Mean±SD: 0.9491 ± 0.0063 |
| ANN - SMOTE | Fold 1 Accuracy: 0.9332  Fold 2 Accuracy: 0.9300  Fold 3 Accuracy: 0.9417  Fold 4 Accuracy: 0.9321  Fold 5 Accuracy: 0.9459  Mean±SD: 0.9366 ± 0.0061 | Fold 1 Accuracy: 0.9449  Fold 2 Accuracy: 0.9258  Fold 3 Accuracy: 0.9374  Fold 4 Accuracy: 0.9416  Fold 5 Accuracy: 0.9427  Mean±SD: 0.9385 ± 0.0068 |
| GDBT - SMOTE | Fold 1 Accuracy: 0.9406  Fold 2 Accuracy: 0.9343  Fold 3 Accuracy: 0.9491  Fold 4 Accuracy: 0.9416  Fold 5 Accuracy: 0.9459  Mean±SD: 0.9423 ± 0.0050 | Fold 1 Accuracy: 0.9449  Fold 2 Accuracy: 0.9406  Fold 3 Accuracy: 0.9491  Fold 4 Accuracy: 0.9427  Fold 5 Accuracy: 0.9384  Mean±SD: 0.9431 ± 0.0037 |
| TabNet - SMOTE | Fold 1 Accuracy: 0.9330  Fold 2 Accuracy: 0.9483  Fold 3 Accuracy: 0.9414  Fold 4 Accuracy: 0.9015  Fold 5 Accuracy: 0.8964  Mean±SD: 0.9241 ± 0.0212 | Fold 1 Accuracy: 0.9296  Fold 2 Accuracy: 0.9321  Fold 3 Accuracy: 0.9457  Fold 4 Accuracy: 0.9066  Fold 5 Accuracy: 0.9041  Mean±SD: 0.9236 ± 0.0159 |

Abbreviations: AdaBoost, Adaptive Boosting; CatBoost, Categorical Boosting; GDBT, Gradient Boosting Decision Tree; LightGBM, Light Gradient Boosting Machine; XGBoost, eXtreme Gradient Boosting; RF, Random Forest; ANN, Artificial Neural Network; ROS, Random Over-Sampling; SMOTE, Synthetic Minority Over-sampling Technique

**Supplementary Table 7. The important variables of LightGBM model**

| Important variables of LightGBM model |
| --- |
| X1, X2, X3, X4, X5, X6, X7, X8, X9, X10, X11, X12, X13, X14, X15, X19, X23, X26, X28, X33, X39, X40, X41, X44, X45, X47, X51, X53, X54, X59, X61, X62, X63, X64, X65, X66, X67, X68, X80, X81, X82, X83, X84, X85, X86, X87, X88, X90, X91, X93, X94, X95, X96, X97, X98, X99, X100, X103, X104, X106, X107, X108, X109, X110, X111, X112, X113, X114, X115, X116, X117, X118, X119, X120, X121, X122, X123, X126, X127, X128, X129, X130, X133, X135, X136, X143, X147, X149, X151, X153, X154, X156, X158, X159, X160, X162, X167, X168, X173, X176, X177, X179, X180, X181, X187, X189, X192, X193, X195, X196, X197, X205, X208, X209, X210, X214, X224, X233, X235, X236, X238, X239, X243, X246, X250, X251, X252, X256, X257, X259, X261, X263, X264, X271, X272, X276, X277, X278, X281, X283, X285, X287, X290, X292, X295, X296, X303, X306, X307, X311, X312, X318, X319, X321, X322, X323, X324, X325, X327, X330, X331, X335, X337, X338, X340, X342, X343, X347, X349, X353, X355, X358, X360, X361, X365, X366, X367, X368, X372, X374, X377, X378, X380, X381, X382, X383, X384, X390, X391, X393, X394, X396, X403, X405, X410, X412, X413, X419, X420, X421 |
